# The Differential Roles for Neurodevelopmental and Neuroendocrine Genes in Shaping GnRH Neuron Physiology and Deficiency

**DOI:** 10.3390/ijms22179425

**Published:** 2021-08-30

**Authors:** Roberto Oleari, Valentina Massa, Anna Cariboni, Antonella Lettieri

**Affiliations:** 1 Department of Pharmacological and Biomolecular Sciences, University of Milan, 20133 Milano, Italy; roberto.oleari@unimi.it; 2 Department of Health Sciences, University of Milan, 20142 Milano, Italy; valentina.massa@unimi.it; 3 CRC Aldo Ravelli for Neurotechnology and Experimental Brain Therapeutics, Department of Health Sciences, University of Milan, 20142 Milano, Italy

**Keywords:** GnRH neurons, congenital hypogonadotropic hypogonadism, Kallmann syndrome

## Abstract

Gonadotropin releasing hormone (GnRH) neurons are hypothalamic neuroendocrine cells that control sexual reproduction. During embryonic development, GnRH neurons migrate from the nose to the hypothalamus, where they receive inputs from several afferent neurons, following the axonal scaffold patterned by nasal nerves. Each step of GnRH neuron development depends on the orchestrated action of several molecules exerting specific biological functions. Mutations in genes encoding for these essential molecules may cause Congenital Hypogonadotropic Hypogonadism (CHH), a rare disorder characterized by GnRH deficiency, delayed puberty and infertility. Depending on their action in the GnRH neuronal system, CHH causative genes can be divided into neurodevelopmental and neuroendocrine genes. The CHH genetic complexity, combined with multiple inheritance patterns, results in an extreme phenotypic variability of CHH patients. In this review, we aim at providing a comprehensive and updated description of the genes thus far associated with CHH, by dissecting their biological relevance in the GnRH system and their functional relevance underlying CHH pathogenesis.

## 1. Introduction

Fertility and reproduction of sexually reproducing species strictly depend on a functional hypothalamic–pituitary–gonads (HPG) axis, which ensures gonadal development, puberty onset and reproductive capacity.

The HPG axis is a neuroendocrine circuit centrally regulated by hypothalamic gonadotropin-releasing hormone (GnRH) neurons, which, in humans, release the GnRH decapeptide in a pulsatile fashion within the pituitary blood portal system to stimulate gonadotrope cells to secrete gonadotropins (i.e., LH and FSH). Once released, gonadotropins reach, through circulation, the gonads, where they induce sex steroid production [1].

Because the neurohormone GnRH is the primary driver of the HPG axis, proper development and function of its producing neurons is required. In this context, several factors finely regulate GnRH neuron physiology by acting at different levels, including GnRH neuron development and differentiation, GnRH synthesis, secretion and action. Accordingly, defects in either GnRH neuron development or function can lead to a pathological condition known as isolated GnRH deficiency or Congenital Hypogonadotropic Hypogonadism (CHH), characterized by incomplete or absent puberty and infertility [2].

This review provides an up-to-date description of all the genes associated with CHH by focusing on their biological roles in GnRH neuron system development, uncovered by experimental studies through in vitro and in vivo models.

## 2. GnRH Neuron Development and Function

GnRH neurons, despite their key role in the control of the HPG axis, consist of a small number of cells (approximately 2000 and 800 cells in primate and rodent adult brains, respectively [3]), which are dispersed in a bilateral continuum throughout the hypothalamus, with most of their cell bodies concentrated in the medial preoptic area (MPOA) [4]. Interestingly, GnRH neurons have also been detected in extrahypothalamic regions, such as olfactory bulbs (OBs), amygdala and hippocampus, but their role in these regions remains unknown [3,5].

### 2.1. GnRH Neuron Development in the Nasal Compartment

The unique feature of GnRH neurons is their embryonic site of origin, which is external to the central nervous system. Specifically, during development, immature GnRH precursor neurons are first detected in the olfactory placode (OP) within the nose, as early as embryonic day (E) 10.5 in mice [4]. The OP gives rise to the nonsensory respiratory epithelium, the sensory olfactory epithelium and the vomeronasal organ (VNO), where cell bodies of olfactory (OLF), vomeronasal (VN) nerves, GnRH neurons and olfactory ensheathing cells (OECs) are contained [6].

GnRH neurons are thought to differentiate in a niche at the border between respiratory and VNO due to the presence of pro-neurogenic signals, including FGF8 and NOG, and neurogenic repressors such as BMP4 [7]. The specification of GnRH neurons remains elusive, but previous lineage tracing and ablation studies suggest that GnRH neurons arise from two precursor populations having ectodermal and neural crest derivation from the vomeronasal organ (VNO) [7,8,9]. Recent studies in mice and human iPSCs highlight the importance of LIM-homeodomain transcription factor ISL1 in the differentiation of ectodermal-derived GnRH neurons [10,11].

Post-mitotic GnRH neurons delaminate from the VNO epithelium, invade the nasal mesenchyme and begin a complex journey towards the basal forebrain [12,13]. For years, the prevailing view was that the migratory route of GnRH neurons was patterned by OLF/VN axons in the nose and by a transient branch of VN axons (i.e., caudal branch) in the forebrain [4,13,14]. However, recent findings proposed that the terminal nerve (TN, also called cranial nerve 0 or the caudal branch of VN nerves) acts as a unique axonal scaffold for GnRH neuron migration [15], which appears to be independent from olfactory system development. Accordingly, the TN projects ventrally and dorsally within the forebrain to target hypothalamic areas, rather than innervating OBs, thus providing support to GnRH neuron migration in the brain parenchyma [3,15]. The migration of GnRH neurons and the patterning of OLF/VN/TN nasal axons are controlled by several molecules, including adhesion molecules (e.g., anosmin-1), neurotransmitters (e.g., GABA), growth factors (e.g., FGF8/FGFR1) and chemotactic cues (e.g., semaphorin signaling members, NTN1, CXCL12) [12,13,16,17,18].

Interestingly, some of the genes implicated in olfactory and GnRH neuron systems have been well studied in mice but the pathogenic variants of such genes have not always been found in humans. This can be due to the existence of genetic differences between rodents and humans, in addition to physiological differences between the two species (i.e., rodents are more dependent than humans on olfaction in their reproductive behavior).

Recently, the study of GnRH neuron migration has been also explored in humans, due to the availability of human fetuses and tissue clearing technologies. These anatomical studies have provided evidence that GnRH neuron development is conserved between rodents and humans. In humans, GnRH neurons begin to emerge in the OP at Carnegie Stage (CS) 16 (gestation week (GW) 5.5), and migration initiates at CS18 (approximately GW 7) and peaks at CS23 (GW8). At around GW12, most of GnRH neurons have already become set in the forebrain [3]. However, although few murine genes have also been found to be expressed in human embryos to date, suggesting the existence of conserved genetic pathways, further studies using human models, such as iPSCs or organoids, will help to confirm their functional relevance and to dissect the molecular mechanisms involved in human GnRH neuron development. These studies may also help to understand why some of the genes found to be mutated in mouse models do not have a human counterpart and vice versa.

### 2.2. GnRH Neuron Development in the Hypothalamus

The GnRH neuron journey terminates in the developing forebrain when GnRH neurons finally detach from their TN guiding fibers, disperse mainly in the hypothalamic MPOA and extend axons towards the median eminence (ME). At E16.5, the first GnRH neuroendocrine axons are observed in the ME and by E17-E18 GnRH neuronal system is functional and starts to activate the HPG axis [19]. The final steps of GnRH neuron development are poorly studied [13], although some molecules regulating GnRH neuron survival and maturation have been identified.

For instance, AXL/TYRO3 [20], NHLH2/NDN [21,22] and, more recently, SEMA3E/PLXND1 [23] signaling pathways have been shown to exert pro-survival effects on GnRH neurons within the MPOA. Similarly, PROK2 and its receptor, PROKR2, have been suggested to regulate maturation/survival of GnRH neurons, because they are both expressed in the MPOA [24].

Further, GnRH neurons acquire a bipolar morphology, with axons extending to the ME and proximal dendrites. Several factors have been proposed to drive neurite extension of mature GnRH neurons, including FGF2/FGFR1 [25,26] NTN1 [27] and SEMA7A/ITGB1 [28]. Recent studies highlighted the peculiarity of GnRH neuron distal processes in sharing the characteristics of both dendrites and axons, which are therefore termed “dendrons” [29]. These unusual structures represent another remarkable feature of GnRH neurons and are believed to be involved in the fine control of GnRH secretion [30,31].

Finally, mature GnRH neurons create complex neuronal networks that integrate a wide variety of internal and external factors to control GnRH secretion, such as steroids, metabolic hormones, stress and the season [32,33]. In recent years, individual components of the neural circuits underlying GnRH secretion began to emerge (reviewed in [34]), of which the most important is the Kisspeptin (Kiss1) neuron afferent population. In the hypothalamus, two distinct Kiss1 neuron populations can be found: one population resides in the anteroventral periventricular nucleus (AVPV) and one in the arcuate nucleus (ARC) [35]. ARC Kiss1 neurons express Dynorphin and Neurokinin B, and are therefore named “KNDy” neurons [36], and, as a result, cooperate in GnRH pulsatile release coordination [37]; in contrast, AVPV Kiss1 neurons mediate estrogen positive feedback and are important to sustain the GnRH pre-ovulatory surge [37].

## 3. Congenital Hypogonadotropic Hypogonadism (CHH)

CHH is characterized by inappropriately low serum concentrations of the gonadotropins, LH and FSH, in the presence of low circulating concentrations of sex steroids that lead to the absence of puberty, infertility and consequent reproductive failure [2]. CHH incidence is uncertain and can vary broadly from 1:4000 [38] to 1:30,000 [39] in the male population, with a prevalence of around 4 to 1 compared to the female population [2,40,41]. Most patients are diagnosed late in adolescence or adulthood as they display arrested or absent puberty, clinical evidence of hypogonadism and incomplete sexual maturation [2]. Adult males with CHH tend to have prepubertal testicular volume (i.e., <4 mL), absence of secondary sexual features (e.g., facial and axillary hair growth, deepening of the voice), decreased muscle mass, diminished libido, erectile dysfunction, and infertility. Adult females have little or no breast development and primary amenorrhea [40,41]. Infant boys with CHH often have micro phallus and cryptorchidism (i.e., undescended testes), thus providing the possibility of an early diagnosis [42,43]. CHH can be considered a chronic condition [44] and may lead to many comorbidities, including psychological disorders [45], osteoporosis [46] and increased risk of metabolic defects (e.g., type II diabetes mellitus) [47]. However, 10–20% of CHH patients exhibit a spontaneous recovery (reversal CHH) [48], although in some cases relapses may be experienced [49].

Clinically, CHH can be solely present with reproductive symptoms (normosmic CHH) or in association with olfaction defects (hyposmia/anosmia), being referred to as Kallmann syndrome (KS) and representing 50% of overall CHH cases [2,38]. KS patients may also exhibit non-reproductive and non-olfactory features, such as bimanual synkinesis, abnormal eye movements, hearing impairment, agenesis of the corpus callosum, unilateral or bilateral renal agenesis, cleft lip or palate, and hypodontia [50]. To increase its phenotypic complexity, CHH may overlap with multisystemic syndromes including CHARGE syndrome, Waardenburg syndrome, Gordon Holmes syndrome, Dandy–Walker syndrome, Hartsfield syndrome, septo-optic dysplasia, combined pituitary hormone deficiency, adrenal hypoplasia and congenital obesity [2,39,51]. In addition, a small subset of patients may present with adult-onset CHH, which is characterized by normal puberty onset and fertility, followed by the disruption of the HPG axis during adulthood [40,52]. Adult-onset CHH is often associated with metabolic disorders and obesity [53]. These conditions may therefore represent acquired cofactors responsible for CHH onset among adult subjects, who are naturally prone to develop a central failure of the gonadal axis, but carry variants in CHH genes that alone cannot result in disease [54].

The phenotypic heterogeneity of CHH is the result of a complex genetic architecture in addition to different patterns of inheritance. To date, pathogenic variants in 54 genes have been identified with X-linked, autosomal recessive and autosomal dominant inheritance [2,41].

In addition to the view of CHH as a monogenic disorder, it is now well demonstrated that CHH can be transmitted with digenic/oligogenic modes of inheritance [55,56,57]. However, up to 50% of CHH patients do not have an identified causative gene [40,41,58].

## 4. Genetics of CHH

**Figure 1:**
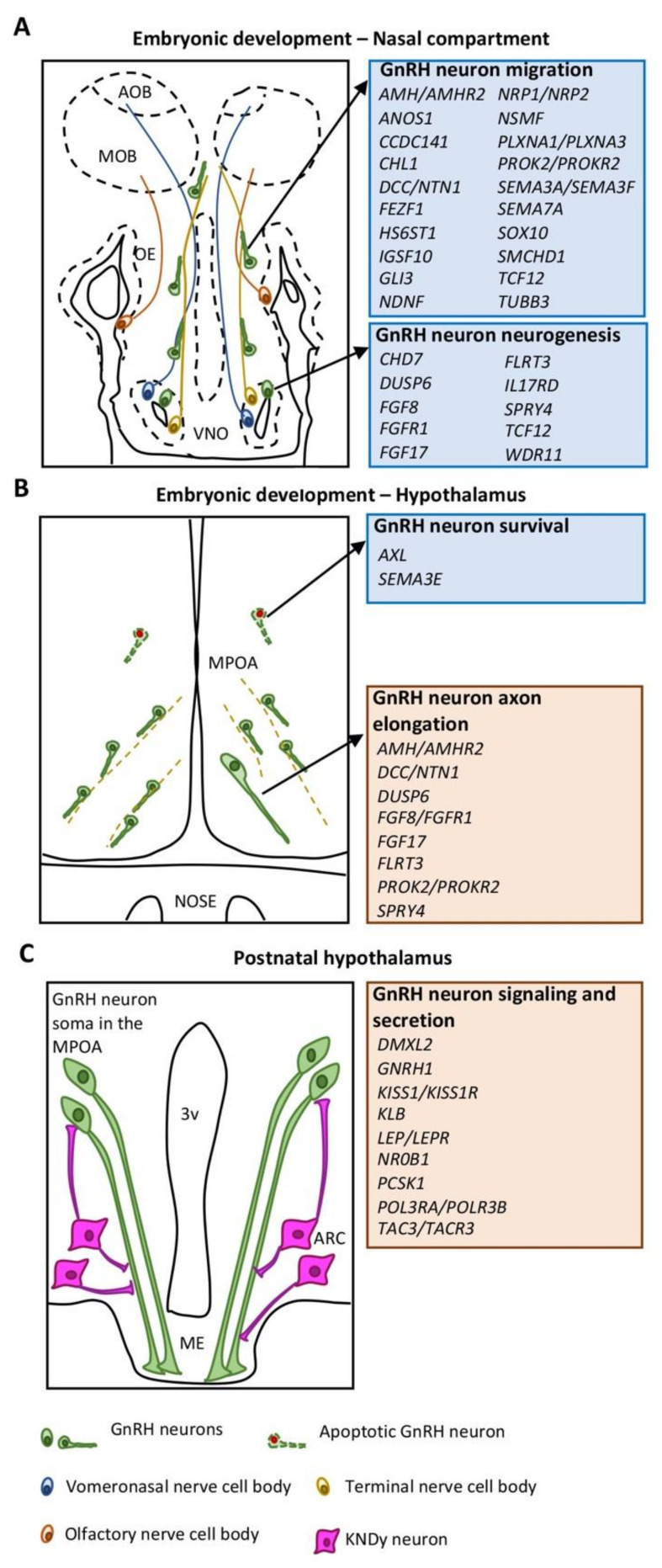
Schematic drawings illustrating the different phases of GnRH neuron development and associated CHH genes. (**A**) Embryonic development of GnRH neurons in the nasal compartment is orchestrated by neurodevelopmental genes (blue box) regulating either the neurogenesis or the migration of GnRH neurons. (**B**) Embryonic development of GnRH neurons in the MPOA of the hypothalamus is controlled by both neurodevelopmental (blue box) and neuroendocrine (red box) genes mainly implicated in GnRH neuron survival and axon elongation, respectively. (**C**) GnRH neuronal function in the hypothalamus is mediated by neuroendocrine genes (red box) controlling GnRH secretion or signaling. Abbreviations: VNO, vomeronasal organ: OE, olfactory epithelium; MOB, main olfactory bulb; AOB, accessory olfactory bulb; MPOA, medial preoptic area; ME, median eminence; ARC, arcuate nucleus; 3v, third ventricle.

Depending on their biological function in the GnRH neuronal system, CHH causative genes can be divided into two main groups: (1) genes implicated in the action/signaling of GnRH in normally developed GnRH neurons (neuroendocrine genes); (2) genes involved in GnRH neuron ontogeny, migration and survival (neurodevelopmental genes). In addition, some genes exert their function in both biological contexts (Figure 1).

Of note, neurodevelopmental genes affecting the development of VNO and its derivatives (i.e., GnRH neurons and TN/VN axons) showed a higher prevalence in CHH cohorts compared to neuroendocrine genes. Hence, *ANOS1*, *CHD7*, *FGF8*/*FGFR1*, *SEMA3A*, *SOX10* and *PROKR2* variants account for ~35–40% of the overall mutated loci underlying CHH [41,59].

Herein, we review the genes found to be implicated in the GnRH system, and whose variants are thus far associated with CHH (Table 1). Most of these genes have been studied by applying experimental models, including mouse cell lines, transgenic rodents and alternative models, such as zebrafish [60,61,62]. Prior to exome sequencing technologies, these models have been instrumental in the prediction of candidate genes that have then been screened for mutations in patients. More recently, new CHH-associated genes have been discovered in human patients due to NGS/high throughput screening, but experimental models have been essential to confirming their functional relevance and the pathogenicity of the mutations.

In this review, we also provide insights into genes that are still not recognized as CHH causative genes but have been described to play a role in GnRH neuron biology and found to be mutated in patients with CHH or overlapping syndromes. The causative CHH genes associated with pituitary development and function (e.g., *FSHB, GATA2, GLI2, GNRHR, HESX1*, *LHB*, *LHX3*/*4*, *OTX2*, *PITX2,* *PROP1* and *SOX2/3*; reviewed in [41,63]) are not discussed in this review.

### 4.1. Neuroendocrine Genes

#### 4.1.1. Gonadotropin Releasing Hormone 1 (GNRH1)

In humans, the hypophysiotropic form of GnRH decapeptide is encoded by *GNRH1* gene (chr 8p21.2) and, although variants in this gene are expected to be disease causing, *GNRH1* variants are rare (2% of normosmic CHH cases) [41]. In 1986, Mason and colleagues demonstrated that *hpg* mice, which completely lack GnRH due to a truncating deletion in *Gnrh1*, are sexually immature and infertile [64]. However, the first loss-of-function variants were only found in 2009 within exons encoding for the GnRH decapeptide [65,66]. A later study also described two novel variants in loci encoding for the mature peptide, suggesting these areas as likely mutational areas [67].

Interestingly, the *GNRH1* variant W16S (rs6185), which is normally associated with delayed puberty onset in females, has been shown to significantly delay the onset of menopause, suggesting a direct role of the HPG axis in menopause timing [68].

#### 4.1.2. Kisspeptin (KISS1) and Kisspeptin Receptor (KISS1R)

*KISS1* (chr 1q32) and its receptor *KISS1R* (chr 19p13.3), which is expressed by most GnRH neurons [69], are essential modulators of GnRH secretion and puberty onset. They participate as a GnRH pulse generator and ensure the pre-ovulatory GnRH surge [19]. Hence, defective KISS1/KISS1R signaling strongly impairs GnRH secretion, and variants in both *KISS1* [70] and *KISS1R* [71,72] genes were accordingly found in normosmic CHH patients.

Functional studies in transgenic mouse models confirmed overall the pivotal role of KISS1/KISS1R signaling in driving pubertal onset and fertility [73]. In particular, mice lacking either *Kiss1r* [72] or *Kiss1* [74] fail to sexually develop. However, mice lacking *Kiss1* display a variable reproductive phenotype compared to mice lacking the receptor counterpart showing a more severe hypogonadal phenotype [75].

In contrast, some studies challenged the idea that KISS1/KISS1R signaling is essential for GnRH release modulation and sexual maturation. Interestingly, mice lacking either *Kiss1* or *Kiss1r* exhibit a residual GnRH activity, suggesting a KISS1/KISS1R-independent mechanism of GnRH secretion [76].

Of note, recent studies provide evidence that developmental defects impairing Kiss1 neuron specification, due to both *Ptf1a* [77] and *Prdm13* [78] deletion, can result in pronounced hypogonadism or delayed puberty onset in mice, respectively.

Finally, it was observed that KISS1 neurons significantly increase in number with aging in both males and females, when levels of circulating sex steroids are lower [79,80]. However, the effects of kisspeptin in aging populations remain largely unexplored.

#### 4.1.3. Neurokinin B (TAC3) and Neurokinin B Receptor (TACR3)

*TAC3* (chr 12q13-q21) and its coupled receptor *TACR3* (chr 4q25) variants were first found due to a homozygosity mapping-driven strategy from consanguineous families [81] and, to date, several mutations have been discovered [82,83]. Neurokinin B is expressed by KNDy neurons in the ARC and contributes to the GnRH pulsatile secretion by positively stimulating KISS1 release from KNDy neurons themselves with autocrine and paracrine mechanisms through TACR3 [36].

In vivo studies only partially recapitulate the CHH phenotype of patients. Both *Tac2* [84], encoding for neurokinin B in mice, and *Tacr3* [85] knockout mice exhibited normal sexual maturation, with the exception of *Tac2^−/−^* females. In addition, *Tacr3*-null mice are mildly hypogonadal, despite normal fertility.

#### 4.1.4. Leptin (LEP) and Leptin Receptor (LEPR)

CHH patients may occasionally display a mild obese phenotype; however, patients affected by normosmic CHH and severe early-onset obesity harbor pathogenic variants in *LEP* (chr 7q31.3) [86] and *LEPR* (chr1p31) [87] genes. The *LEP* gene encodes for leptin, which is a 167 amino acid cytokine produced by white adipose tissue that exerts an anorexigenic role in the hypothalamus. Leptin positively modulates GnRH secretion, providing a link between metabolic status and reproduction [88], although its action is not direct on GnRH neurons, which do not express LEPR [89]. Indeed, the permissive role of leptin on GnRH secretion is thought to be due to a subset of LEPR-expressing KNDy neurons [35]. It has been alternatively proposed that leptin may inhibit neuropeptide Y and, in turn, promote POMC/*α*-MSH (proopiomelanocortin/melanocortin) secretion, which have a negative and positive effect on GnRH release, respectively [90]. Mice lacking leptin (*ob/ob* mice), display obesity and hypogonadism, which can be restored by leptin treatment [91]. By comparison, neuron-specific loss of *Lepr* leads to obesity and infertility [92].

#### 4.1.5. Proprotein Convertase Subtilisin/Kexin Type 1 (PCSK1)

Also known as neuroendocrine convertase 1, PCSK1 is required for the processing of large precursor pro-hormones such as POMC, insulin and glucagon, and, therefore, for the release of active peptides [93]. To date, the role of PCSK1 in the HPG axis has not been fully elucidated. However, *PCSK1* (chr 5q15-q21) variants were found in patients with normosmic CHH and obesity [94,95,96,97]. Interestingly, these variants were found to affect the catalytic domain, strengthening the idea that *PCSK1* variants lead to an impaired pro-hormone processing.

#### 4.1.6. β-klotho (KLB)

In addition to its well-described role in GnRH neuron ontogenesis, FGFR1 has been proposed to exert other reproductive roles, including the regulation of GnRH neuron homeostasis and metabolism [98]. However, because its main ligand FGF8 is restrictively expressed during embryogenesis [99], researchers have proposed that FGF21 is a potential alternative ligand for FGFR1 [100]. FGF21 acts centrally as a metabolic regulator by acting through FGFR1 and its obligate co-receptor KLB.

*KLB* (chr 4p14) was first recognized as a senescence-related gene, acting as an inhibitor of aging and linked to the onset of age-related disorders [101]. Consistently, *Klb*-null mice exhibit a syndrome resembling human aging, characterized by short lifespan, infertility, arteriosclerosis, skin atrophy and osteoporosis [102].

In respect to GnRH neurons, KLB expression is barely detectable at embryonic stages but increases in mature GnRH neurons. *Klb*-null mice have normal GnRH neuron development but display delayed puberty and subfertility. In addition, in vitro experiments in immortalized mature GnRH neurons (GT1-7 cells) showed that FGF21 promotes GnRH neuron neurite extension and GnRH secretion. Interestingly, although pathogenic *FGF21* variants have not been described to date, heterozygous *KLB* variants have been found in normosmic CHH patients [100].

#### 4.1.7. Nuclear Receptor Subfamily 0 Group B Member 1 (NR0B1)

Pathogenic variants in *NR0B1* (or Dosage-sensitive sex reversal, adrenal hypoplasia critical region, on chromosome X gene, DAX1; chr Xp21.2) are usually causative of X-linked adrenal hypoplasia congenita [103]. In addition to its expression in the adrenal axis, DAX1 has additional functions in the development of the organs forming the HPG axis [104,105]. Accordingly, a reduction in GnRH release was shown by in vitro assay [106], whereas a hypogonadal and infertile phenotype was described in male mice lacking *Dax1* [107]. The copresence of X-linked congenital adrenal hypoplasia and hypogonadotropic hypogonadism in patients harboring *DAX1* variants was firstly described in 1994 [108] and, to date, few other cases have been reported. In addition, rare cases of female carriers of *DAX1* variants were reported to develop delayed puberty phenotype, without any adrenal feature, probably as a result of the non-random inactivation of the X chromosome [109]; however, detailed molecular investigations have not been undertaken.

#### 4.1.8. Dmx-like 2 (DMXL2)

*DMXL2* (chr 15q21.2) encodes for the vesicular protein rabconnectin-3α, which is expressed on exocytosis vesicles of the GnRH neuron axon terminals within the ME [110]. Further, it critically determines the capacity of GnRH neurons to release GnRH in response to Kiss1 neuron stimuli and is also involved in the pruning of GnRH neuron dendrites [111]. Accordingly, *Dmxl2* heterozygous mice exhibit delayed puberty onset and a low fertility rate [110]. Patients carrying *DMXL2* variants are affected by normosmic CHH, central hypothyroidism, peripheral demyelinating sensorimotor polyneuropathy, mental retardation and profound hypoglycemia, consistent with polyendocrine deficiencies and polyneuropathies [110].

#### 4.1.9. Patatin-like Phospholipase Domain Containing Protein (PNPLA6), OTU Deubiquitinase 4 (OTUD4), Ring Finger Protein 216 (RNF216), STIP1 Homology and U-Box Containing Protein 1 (STUB1)

*PNPLA6* (chr 19p13.2) encodes for a phospholipase involved in de-esterification of membrane phosphatidylcholine and is widely expressed throughout the mouse brain. Loss-of-function of *Pnpla6* in mice is lethal in the early embryonic stage [112]. *PNPLA6* variants have been found in patients affected by Gordon Holmes and Boucher–Neuhauser syndromes, which are characterized by normosmic CHH and neurodegenerative cerebellar ataxia [113].

Gordon Holmes syndrome was also diagnosed in patients carrying pathogenic variants in ubiquitination pathway genes *OTUD4* (chr 4q31.21), *RNF216* (chr 7p22.1) [114] and *STUB1* (chr 16p13.3) [115]. In agreement, male *Rnf216^−/−^* [116] and *Stub1^−/−^* [115] mice are hypogonadal and display impaired spermatogenesis, whereas females are not affected by *Rnf216* loss [116]. In vitro experiments also support a direct role of RNF216 in GN11 cell migration [117]. No murine studies are available for OTUD4, but its biological role has been validated through morpholino-mediated knockdown in zebrafish [114].

#### 4.1.10. Polymerase III, RNA Subunit A/B (POLR3A/POLR3B)

Polymerase III is an enzyme involved in the transcription of small untranslated RNAs and is required for the regulation of essential cellular processes [118]. This enzyme is composed of many subunits including *POLR3A* (chr 10q22.3) and *POLR3B* (chr 12q23.3) subunits, which constitute the enzyme’s catalytic center. Variants in these two subunits are associated with hypomyelinating leukodystrophy and a clinically overlapping syndrome presenting with cognitive dysfunction, cerebellar features, dental abnormalities (4H syndrome) [119] and/or normosmic CHH [120].

Both *Polr3a* homozygous knock-in and heterozygous knock-in/knock-out mice for G672E substitution are viable and able to reproduce [121], whereas the *Polr3b* variant R103H is embryonically lethal suggesting that missense mutations in *Polr3a* and *Polr3b* can variably impair mouse development and Pol III function [122].

### 4.2. Neurodevelopmental Genes

#### 4.2.1. Anosmin 1 (ANOS1)

*ANOS1* (chr Xp22.3) was the first KS gene to be discovered in 1991 [123] and several mutations have been identified to date, encompassing 10–15% of overall X-linked KS cases [41]. In addition, in 1989, a single human fetus carrying a Xp22.3 locus deletion was analyzed. Immunohistochemical experiments revealed OLF/VN nerves tangle at the cribriform plate level with GnRH neurons trapped inside, thus failing to reach the hypothalamus [124]. Anosmin-1 is an extracellular matrix glycoprotein of 680 amino acids characterized by four fibronectin-like type III repeats, homologous to cell adhesion molecules, and several predicted heparan sulphate proteoglycan (HSPG) binding regions. Consistent with its structure, anosmin-1 is able to link cell membranes to the regulation by extracellular HSPG of several processes, including neural cell adhesion and axonal migration [125,126].

Due to the lack of orthologue genes in rodents, the functional validation of the direct effect of anosmin-1 on GnRH neurons was provided in 2004 by Cariboni and colleagues. They demonstrated that wild-type but not mutant anosmin-1-enriched media produced a chemotactic response on immature migrating GnRH neurons (GN11 cells) [127] in a Boyden chamber assay [128].

Further, *ANOS1* variants are often associated with other developmental defects, such as renal agenesis, midline defects, hearing impairment and synkynesia [58,129], suggesting anosmin-1 is also involved in other developmental processes. Consistently, anosmin-1 expression was detected in the brain, kidney and facial mesenchyme [130].

Interestingly, X-linked *ANOS1* gene has been described as one of the genes with the strongest evidence for tissue-specific escape [131], supporting the concept that the higher prevalence of the disease in males may be explained by gender differences in anosmin-1 dosage [132].

#### 4.2.2. Heparan Sulphate 6-O Sulfotransferase 1 (HS6ST1)

Pathogenic variants of *HS6ST1* (chr 2q14.3) were found in both KS and normosmic CHH patients [133]. HS6ST1 is an enzyme that catalyzes the specific O-sulphation of HSPG, an important component of the extracellular matrix [134]. HSPG are involved in cell migration, interacting with cell adhesion proteins and cell–cell communication, and regulating gradients of many soluble factors, such as FGFs and VEGFs [135]. Thus, it appears plausible that human *HS6ST1* variants can contribute to GnRH neuron development by compromising signaling through one or more of these pathways. Accordingly, it was demonstrated in *C. elegans* that HS6ST1 can interact with both ANOS1 and FGFR1 orthologue genes [133].

Given the biological importance of functional HSPG, mice lacking *Hs6st1* are embryonically lethal [136]. However, it has been recently reported that heterozygous *Hs6st1* mice exhibited delayed puberty onset [137].

#### 4.2.3. NMDA Receptor Synaptonuclear Signaling and Neuronal Migration Factor (NSMF)

NSMF, also known as nasal embryonic LHRH factor (NELF), is a guidance cue essential for OLF/VN patterning. Its expression peaks at around E12.5–14.5 mainly on the membrane surfaces of both GnRH neurons and nasal axons; thus, an homophilic interaction between the two cell types may be suggested [138]. Knockdown of *Nsmf* in both mice and zebrafish led to abnormal migration of GnRH neurons and defective OLF/VN nerve patterning to the OB [138,139], suggesting that NSMF is a highly conserved factor, playing pivotal roles in the nasal axon patterning and GnRH neuron migration. Phenotypic characterization of *Nsmf*-null mice surprisingly highlighted a sexual dimorphic response to Nsmf loss, with female mice exhibiting a more severe reproductive phenotype and a reduced number of GnRH neurons in the hypothalamus compared to males [140].

Variants in *NSMF* (chr 9q34.3) have been found both in KS and normosmic CHH patients, primarily in an oligogenic pattern of inheritance [141].

#### 4.2.4. AXL Tyrosine Kinase Receptor (AXL)

The tyrosine kinase encoded by the *AXL* gene is a member of the Tyro3-Axl-Mer (TAM) receptor tyrosine kinase subfamily, involved in many physiological processes including cell survival, cell proliferation, migration and differentiation [142]. Analysis of both tyrosine kinase receptor *Axl-* and *Tyro3*-null mice showed delayed puberty onset and partial loss of GnRH neurons in the hypothalamus, due to increased apoptosis of a subset of GnRH neurons already set in the MPOA [20]. These findings were subsequently corroborated by the identification in a single study, to date, which reported *AXL* (chr 19q13.2) variants in KS and normosmic CHH patients [143].

#### 4.2.5. FEZ Family Zinc Finger 1 (FEZF1) and Coiled-Coil Domain Containing 141 (CCDC141)

*FEZF1* (chr 7q31.32) is a zinc finger transcription factor that regulates neurogenesis and neuronal cell fate, especially within the OP [144]. Due to advances in sequencing technologies and by applying autozygosity mapping, Kotan and colleagues identified two probands from consanguineous families carrying pathogenic variants of *FEZF1* and affected by KS [145]. Notably, one of the probands also carried a second pathogenic variant lying on *CCDC141* (chr 2q31.2), which encodes for a cytoskeletal scaffolding protein with a role in cellular motility. Further genetic screenings of large CHH patient cohorts confirmed the presence of *CCDC141* variants in KS patients [146,147].

Murine studies validated the crucial roles exerted by both FEZF1 and CCDC141 during GnRH neuron migration. Specifically, *Fezf1^−/−^* mice lack OB connectivity with FEZF1-expressing nasal axons that fail to cross the cribriform plate and GnRH neurons stacked in the nasal compartment [148]. By comparison, CCDC141 is expressed on nasal axons and GnRH neurons themselves, and its knockdown in nasal explants selectively decreases GnRH neuron motility [146].

#### 4.2.6. Neuron-Derived Neurotrophic Factor (NDNF)

*NDNF* (chr 4q27) encodes for a secreted neurotrophic factor that is involved in many neurodevelopmental processes (migration, growth, survival and neurite outgrowth) [149]. Four different *NDNF* pathogenic variants were recently identified by an integrated analysis comprising whole exome sequencing in a cohort of 240 CHH European patients and bioinformatic tools [150]; however, none of these was then confirmed in a different cohort of 60 Japanese patients [151]. NDNF is highly expressed during murine embryogenesis in the same regions of GnRH neuron development. Loss-of-function studies, both in vitro and in vivo, demonstrated that the lack of this neurotrophic factor strongly impairs GnRH neuron migration and olfactory innervation, supporting a role for NDNF in GnRH neuron biology and disease [150].

#### 4.2.7. WD Repeat Domain 11 (WDR11)

The transcription activator *WDR11* (chr 10q26.12) was first found to be mutated in patients affected by normosmic CHH and KS in 2010, and a synergistic relationship between WDR11 and homeodomain transcription factor EMX1, involved in the development of olfactory neurons, was described [152]. More recently, *Wdr11*-null mice have been generated and their phenotypic characterization provided insights into the role of WDR11 in ciliogenesis and the hedgehog signaling pathway [153]. Specifically, *Wdr11^−/−^* mice exhibited a reduced number of hypothalamic GnRH neurons and infertility, in addition to holoprosencephaly and pituitary dysgenesis. The latter is in agreement with combined pituitary hormone deficiency, which was also reported in patients carrying *WDR11* variants [154].

#### 4.2.8. Semaphorins and Receptors

1. Semaphorin 3A and 3F (SEMA3A/3F), Neuropilin 1 and 2 (NRP1/2), Plexin A1 and A3 (PLXNA1/A3)

The involvement of SEMA3A (chr 7q21.11) in the GnRH neuron system was first described in knockout mouse models [155]. In this work, it was shown that SEMA3A, by binding to its co-receptors NRP1/2 [156], patterns OLF/VN/TN fibers granting the axonal scaffold for GnRH neuron migration. Accordingly, misrouted nasal axons, associated with a decreased number of GnRH neurons in the hypothalamus blocked at the level of the cribriform plate, were described with different shades of defects in mice lacking *Sema3a* or SEMA3A-signaling genes *Nrp1/2* [155,157,158].

NRPs lack an intracellular signaling-transducing domain; therefore, a class A plexin is typically required to mediate SEMA3A signals [156]. It was recently described that *Plxna1^−/−^/Plxna3^−/−^* double mutant mice phenocopied GnRH neuron and olfactory defects observed in *Sema3a*-null mice, included hypogonadism in both sexes [159]. In contrast, single *Plxna1* loss only mildly affects the GnRH neuron and olfactory systems in mice [160]. Hence, these results strongly suggest that PLXNA1 and PLXNA3 serve as the main receptors for properly transducing the SEMA3A signal in GnRH neuron and nasal axon migration.

In agreement with the mouse model phenotype, pathogenic variants were first found in *SEMA3A* [158,161,162] and, subsequently in *NRP1*, *NRP2* and *PLXNA1* in normosmic CHH/KS patients [160,163].

Further, variants in *SEMA3F* (chr 3p21.31) and its preferential receptor *PLXNA3* (chr Xq28) were recently found in 15 patients belonging to 11 independent families with normosmic CHH/KS [164]. *PLXNA3* is believed to undergo random X-inactivation [165]. As a result, *Plxna1^−/−^*; *Plxna3^+/−^* female mice exhibited an intermediate severity in the GnRH phenotype [159]. However, human studies are limited to a single report, which highlights a higher prevalence of normosmic CHH/KS in males [164].

Kotan and colleagues also detected SEMA3F expression in human embryos alongside VN and TN axons, also suggesting a role for this semaphorin signaling in human puberty onset and reproduction [164]. However, phenotypic analysis of *Sema3f^-−/−^* mice previously demonstrated that SEMA3F is dispensable for nasal axon patterning and GnRH neuron migration [155].

Finally, a rare single variant in *CHL1* (cell adhesion molecule L1-like, chr 3p26.3), acting as co-receptor for NRP1-SEMA3A signal transduction, was recently described in one pedigree presenting CHH and anosmia. Chen et al. revealed a role of CHL1 in GnRH neuron migration and survival, showing by in vitro studies an impaired ability of migration and the increase in necroptosis in CHL1-mutant cells [166]. *Chl1*-null mice also exhibited abnormal OLF axon guidance but the reproductive phenotype was not evaluated [167].

2. Semaphorin 3E (SEMA3E)

To date, *SEMA3E* (chr 7q21.11) variants have only been reported in two studies: in two brothers with KS, alongside *CHD7* mutation [23], and in a normosmic CHH patient in combination with a *PLXNA1* variant [163]. Although SEMA3E is known to modulate axonal growth through PLXND1 [168,169], in the GnRH neuronal system it promotes survival in PLXND1-expressing GnRH neurons once they have reached the hypothalamus. Therefore, dysfunctional SEMA3E signaling via PI3K results in fewer GnRH neurons surviving in the hypothalamus [23].

In addition, *SEMA3E* mutations have also been reported in a single CHARGE syndrome patient [170], supporting the possible genetic interactions between semaphorins and *CHD7*, the main gene mutated in CHARGE syndrome [171].

Finally, *SEMA3E* variants were found to be enriched in patients affected by mild forms of CHH and adult-onset CHH. These findings support a common genetic architecture underlying these pathological conditions [54].

3. Semaphorin 7A (SEMA7A)

SEMA7A is the only glycosyl-phosphatidylinositol-anchored member of the semaphorin family and signals through either PLXNC1 or ITGB1 (β1-integrin) receptors [156]. A lack of SEMA7A signaling through β1-integrin, which is expressed by GnRH neurons, was previously associated with a decreased migration and neurite extension of GnRH neurons. Together, these developmental defects resulted in a reduced gonadal size and subfertility in both *Sema7a-* and *Itgb1*-null mice [28,172].

Subsequently, mutations found in KS patients have also confirmed the essential role of *SEMA7A* (chr 15q22.3-q23) in humans [162].

#### 4.2.9. Immunoglobulin Superfamily Member 10 (IGSF10)

In mouse embryos, the expression pattern of secreted immunoglobulin superfamily member *Igsf10* showed a ventral to dorsal gradient between the VNO and OBs. Therefore, IGSF10 was postulated to directly act on migrating GnRH neurons, similar to other chemotactic cues (e.g., SEMA4D, CXCL12) [173]. However, receptor(s) for secreted IGSF10 are still unknown and mice lacking *Igsf10* were not available. Hence, the role of IGSF10 in GnRH neuron migration was determined due to gene knockdown experiments in zebrafish and immortalized GN11 cells [173]. *IGSF10* (chr 3q25.1) was found to be mutated in patients affected by normosmic CHH [173].

#### 4.2.10. Chromodomain Helicase DNA Binding Protein 7 (CHD7)

*CHD7* (chr 8q12.2) gene encodes for a chromatin remodeler protein whose expression was detected in the undifferentiated neuroepithelium and neural crest-derived mesenchyme and, later in the development, in olfactory epithelia, OBs and nasal nerves [174,175,176,177]. Variants in *CHD7* are mainly associated with CHARGE syndrome, presenting coloboma, heart defects, atresia of choanae, retarded growth, genital defects, and ear abnormalities as variable clinical traits [178]. Studies in a human fetus with CHARGE syndrome showed arhinencephaly (OB agenesis) with an absence of GnRH neurons in the forebrain [179]. Accordingly, *Chd7*-deficient mice display smaller OB, reduced number of nasal axons, defective sense of smell, reduced hypothalamic GnRH neurons, hypogonadism and impaired pubertal timing [175,176,177].

Missense variants in *CHD7*, alone or in combination with other CHH-causative genes (e.g., *SEMA3E*, *SEMA3A*, *FGFR1* and *GNRHR*), have also been found in patients with KS [23,180,181,182]. However, the precise role of CHD7 in the ontogeny of the GnRH neurons is still under active investigation. To date, it has been demonstrated that CHD7 modulates SEMA3A expression, thus playing a role in both neural crest cell guidance and in the right patterning of TN and, therefore, in GnRH neuron development [183,184]. Moreover, the double hemizygous loss of *Chd7* and *Plxnd1* in mice worsens the GnRH neuron phenotype compared to single heterozygous mutants [23].

#### 4.2.11. Sex-Determining Region (SRY) Box 10 (SOX10)

*SOX10* (chr 22q13.1) encodes for a transcription factor that controls neural crest cell development. In particular, it regulates neural crest-derived olfactory cells, such as OECS, that provide essential mechanical and chemical support for OLF/VN nerves and GnRH neurons [185]. *Sox10*-null mutant mice showed a strong reduction of OECs and an aberrant pathfinding of the nerve fibers leading to an impaired GnRH neuron migration and defects of OBs [186]. Accordingly, *SOX10* (chr 22q13.1) variants are responsible for approximately one-third of KS cases with deafness [186].

However, defective SOX10 signaling can also result in complex syndromes (i.e., Waardenburg syndrome and Hirshsprung disease), presenting phenotypic features that may be attributed to defective neural crest cell specification in other developmental contexts [187,188].

#### 4.2.12. Structural Maintenance of Chromosomes Flexible Hinge Domain Containing 1 (SMCHD1)

*SMCHD1* (chr 18p11.32) encodes for an epigenetic repressor that is fundamental for X chromosome inactivation [189] and was associated with rare forms of muscular dystrophy (fascioscapulohumeral muscular dystrophy type 2). In 2017, two independent screenings of patients affected by congenital arhinia, an extremely rare condition leading to absence of the nose and occasionally other craniofacial defects, identified *SMCHD1* variants as its major drivers. In the same studies, the authors found that most patients carrying *SMCHD1* variants presented a clinical phenotype consistent with Bosma arhinia microphthalmia syndrome (BAMS), characterized by the triad arhinia, ocular defects and CHH [190,191]. More recently, Delaney and colleagues specifically evaluated the co-occurrence of CHH and arhinia, providing evidence that most patients exhibited clinical and/or biochemical signs of GnRH deficiency. However, a small portion of female patients had normal breast development and menstrual cycles, suggesting a fully intact reproductive axis [192]. These findings, together with previous studies in arhinic mice [15], indicate that the main olfactory system may not be necessary for the migration of GnRH neurons.

Arhinia is believed to be caused by defective placodal formation and, accordingly, SMCHD1 expression has been reported in the nasal placode of mouse embryo as early as E9.5 [191]. However, complete loss-of-function of *Smchd1* in mouse produces hypomethylation, which causes female-specific embryonic lethality, this hampering functional studies in mice [189]. Experiments in zebrafish demonstrated that morpholino-mediated *smchd1* knockdown was sufficient to recapitulate the BAMS phenotype, which was rescued by co-injection of full-length human wildtype *SMCHD1* transcript, but not with mRNA containing arhinia variants [190].

#### 4.2.13. Transcription Factor 12 (TCF12)

TCF12 (chr 15q21.3) is a member of the basic helix-loop-helix (bHLH) transcription factors subfamily and *TCF12* haploinsufficiency causes premature cranial suture fusion, leading to a rare developmental disorder known as craniosynostosis [193]. A recent report highlighted the presence of *TCF12* variants in 13 individuals from 12 KS pedigrees, with few cases also presenting with craniosynostosis, implicating a possible genotype–phenotype correlation [194].

Early expression studies in mouse embryos showed a strong expression of *Tcf12* transcript in neurogenic areas [195] and *Tcf12*-null mice developed exencephaly with a high percentage of postnatal death [196]. Thus, alternative functional experiments in zebrafish demonstrated that loss of *tcf12* reduced the axonal length of the terminal nerve and impaired GnRH neuron patterning [194]. Further, a bioinformatic screening revealed *STUB1*, a gene previously associated with CHH, as a putative *TCF12* interactor gene. The overexpression of *stub1* in *tcf12* mutant zebrafish is able to restore the GnRH neuron phenotype, strongly supporting the role of TCF12 in the GnRH system [194].

#### 4.2.14. Gli-Kruppel Family Member 3 (GLI3)

*GLI3* (chr7p14.1) encodes as a zinc finger transcription factor that functions as a transcription modulator in the hedgehog signaling pathway.

A large targeted sequencing of 261 candidate genes revealed *GLI3* as a putative CHH causative gene in a normosmic CHH/KS cohort of patients [197]. Human *GLI3* variants are commonly associated with Greig cephalopolysyndactyly (GCPS) and Pallister–Hall syndromes, with a subset of patients also displaying neonatal hypogonadism (i.e., micropenis and undescended testes) [198]. However, the role of GLI3 in GnRH neuron development has only recently been determined and a pathogenic *GLI3* variant has been reported in a patient affected by KS and GCPS [199].

Consistent with a role in the GnRH neuronal system, mice lacking *Gli3* exhibit decreased neurogenesis of vomeronasal neurons and a defective formation of OECs within the nose, resulting in normal GnRH neuron ontogeny but impaired migration [199]. This work also strengthens the idea that OECs are necessary for GnRH neuron migration, by adding to *SOX10* gene a novel OEC-related gene implicated in CHH pathogenesis.

#### 4.2.15. Tubulin Beta 3 Isotype III (TUBB3)

TUBB3 (chr 16q24.3) is among the different tubulin isotypes that constitute microtubules and is required for axon guidance, maturation and maintenance. Variants in *TUBB3* were previously found in Congenital fibrosis of the extraocular muscles CFEOM [200], but the specific *TUBB3* E410K missense mutation has been recently associated with a more complex syndrome that, in addition to CFEOM, also shows KS and additional clinical features. Subjects present a GnRH deficiency phenotype at puberty and olfactory bulb dysgenesis, and male subjects show microphallus/cryptorchidism as neonates [201].

A recent study has shown that disease-associated variants in the *TUBB3* gene can impair netrin-1 signaling by altering netrin-1-mediated axon repulsion/attraction in the developing nervous system [202]. However, *Tubb3^−/−^* mice are viable and do not show any apparent neuroanatomical or behavioral defects, albeit their microtubule appears to be less dynamic [203].

### 4.3. Neurodevelopmental and Neuroendocrine Genes

#### 4.3.1. Fibroblast Growth Factor 8 (FGF8) and Fibroblast Growth Factor Receptor 1 (FGFR1)

*FGF8* (chr 10q24) encodes for a member of the FGF family, involved in the control of cell proliferation, migration and differentiation during embryonic development [204]. FGF8, coupled to its canonical receptor FGFR1 (chr 8p11.22-p11.23), regulates the patterning of many tissues, including the brain [205] and the olfactory system [206].

In the GnRH neuron system, FGF8/FGFR1 signaling has been found to be involved at different levels. First, GnRH neuron neurogenic niches in the OP are defined by pro-neurogenic signals such as FGF8 and TGF-β factor antagonists (e.g., Noggin), in conjunction with neurogenic repressors signals such as BMP4 [7]. Defective Fgf8 expression determined the complete absence of GnRH neurons in a hypomorph mouse model [207,208], whereas loss of *Fgfr1*, which is expressed by GnRH neurons, only severely reduced their number [209]. However, the role of FGF8 in the establishment of neurogenic niches appears to be indirect, as its loss results in an aberrant expansion of BMP4-expressing facial mesenchyme [210]. Second, FGFR1 and FGF8 also control neurite extension toward the ME of the GnRH neuron in the MPOA [25,26].

In agreement with this, autosomal dominant variants in both genes are frequent (10% of overall cases) in normosmic CHH/KS patients [132,207,211]. Patients with defective FGF8/FGFR1 signaling may additionally display cleft lip, corpus callosum aplasia, ear and finger abnormalities (e.g., polydactyly) and dental agenesis [41,129]. In addition, the FGFR1-dependent CHH phenotype is partially overlapping with syndromes such as Hartsfield syndrome, septo-optic dysplasia and split-hand/foot malformation [2,212,213]. Notably, all the affected tissues are positive for FGF8 lineage tracing, as reported by Forni and colleagues [210].

#### 4.3.2. FGF Signaling Genes

FGF17 (chr 8p2.3) has been identified as an alternative FGFR1 ligand based on high sequence identity and co-expression with FGF8. Recently, variants in *FGF17* gene have been reported in normosmic CHH/KS patients, showing other abnormalities consistent with Dandy–Walker malformation [214].

*IL17RD* (Interleukin 17 receptor D; chr 3p14.3) has been identified as a KS causative gene due to large-scale protein–protein interaction analysis, based on the idea that in addition to FGFR1/ANOS1/HS6ST1, other FGF signaling-related proteins may be implicated in CHH pathogenesis coupled with severe hearing loss [214].

Similarly, additional variants in FGF signaling pathway genes were found in a CHH cohort. DUSP6 (Dual Specificity Phosphatase 6; chr 12q21.33) and SPRY4 (Sprouty RTK Signaling Antagonist 4; chr 5q31.1) are repressors of the FGF8/FGFR1-activated MAPK cascade and pathogenic variants were found in patients with either normosmic CHH and KS together with hearing loss [214]. *SPRY4* pathogenic variants also characterized the genetic background of adult-onset CHH [54].

In contrast, *FLRT3* (Fibronectin Leucine Rich Transmembrane Protein 3; chr 20p12.1) is a transmembrane protein that mediates cell adhesion and receptor signaling, and was found to be mutated in KS patients [214].

No murine or functional studies of identified variants have been performed for these genes, but a recent study expanded the genotypic and phenotypic spectra of *DUSP6*, *IL17RD* and *SPRY4* in CHH [215].

#### 4.3.3. Anti-Mullerian Hormone (AMH) and Anti-Mullerian Hormone Receptor 2 (AMHR2)

AMH (chr19p13.3) is a TGF-b family member and exerts its function by binding to its specific receptor (AMHR2) [216]. In addition to its well-known role in sex differentiation and gonadal functions [217,218], the AMH contribution in the GnRH neuron system was only recently reported. It has been shown that AMHR2 is expressed by GnRH neurons during both the fetal and postnatal periods [219], and AMH has been detected during GnRH migration, both in mice and human fetuses [220]. Specifically, AMH appears to promote both GnRH neuron migration [220,221] and GnRH secretion [219]. In addition, the disruption of AMHR2 signaling in vivo causes a defective GnRH migration consequent to VN/ TN misrouted projections, resulting in a reduced number of GnRH neurons in the adult brain and an altered fertility [220].

To date, only four *AMH* or *AMHR2* heterozygous variants have been described in a cohort of 136 CHH patients, although the experimental evidence supports the fact that the perturbation of the AMH/AMHR2 pathway can underlie CHH pathogenesis [220].

#### 4.3.4. Prokineticin 2 (PROK2) and Prokineticin Receptor 2 (PROKR2)

*PROK2* (chr 3p13) gene encodes for a small peptide called prokineticin 2, which interacts with its cognate receptor PROKR2 (chr 20p12.3) to regulate olfactory system development and neuronal progenitor differentiation. In mouse embryos, PROK2 is expressed in the OB and acts as a chemoattractant for nasal nerves expressing PROKR2 [222]. Mice lacking either *Prok2* [24] or *Prokr2* [223] display OB hypoplasia and hypogonadism, with GnRH neurons unable to reach the hypothalamus, thus resembling the human KS phenotype. Interestingly, PROKR2-expressing cells were present both in the nasal compartment and in the MPOA in close relationship with GnRH neurons [24], raising the possibility that PROK2/PROKR2 signaling, in addition to its well-described role in GnRH neuron migration, may also be involved in late developmental phases (e.g., survival function).

Pathogenic variants of *PROK2* and *PROKR2* were first described in patients with KS and additional non-reproductive features (fibrous dysplasia, sleep disorder, synkinesia and epilepsy), but also in siblings with normosmic CHH [24,224,225]. However, heterozygous variants of *PROKR2* do not show a genotype–phenotype correlation, with the majority of these variants being classified as benign or of uncertain significance according to American College of Medical Genetics and Genomics guidelines [226].

#### 4.3.5. Deleted in Colorectal Cancer (DCC) and Netrin 1 (NTN1)

DCC and NTN1 roles in the guidance of GnRH neuron migration were first reported in the early 2000s. Specifically, the chemoattractant NTN1 patterns the extension of DCC-expressing nasal axons and, in particular, of TN [227]. In agreement with this, *Dcc^−/−^* [227] and *Ntn1^−/−^* [228] mouse embryos showed abnormal TN projections and misrouted GnRH neurons that failed to reach the MPOA. In a later study, Low et al. detected NTN1 and DCC in the embryonic MPOA, with the latter expressed on GnRH neurons [27]. They demonstrated that NTN1 selectively stimulates the extension of GnRH neuron neurites towards the ME where they release GnRH, highlighting a dual role of NTN1/DCC signaling in GnRH neuron migration and neuritogenesis [27].

Pathogenic variants in *DCC* and *NTN1* were identified 10 years later due to a structure-based approach, by searching for variants in genes characterized by fibronectin type 3 domains similar to ANOS1. Patients carrying *DCC* and *NTN1* variants were affected by KS, obesity and ear abnormalities [229].

### 4.4. Additional Genes

In addition to the variants described above, some genes have been found to be mutated in normosmic CHH/KS patients; however, their role in the GnRH system is still controversial.

*SRA1* missense variants were recently found in three families, in a cohort of normosmic CHH patients, alone or in combination with the *PNPLA6* variant [230]. However, the analysis of mice lacking *Sra1* did not provide information on its role in the GnRH neuron system, making it difficult to label it as a CHH gene.

Variants in the *TBX3* gene, which encodes for a member of the T-box transcription factors, are associated with ulnar–mammary syndrome, whose symptoms include hypogonadism, delayed puberty, ulnar ray defects and hypoplasia of nipples [231]. Heterozygous variants of *TBX3* have also been recently described in two unrelated families with normosmic CHH and pituitary hypoplasia [232]. However, the role of TBX3 in GnRH neuron physiology has not yet been elucidated. *TBX3* is expressed in the developing and adult arcuate nucleus of the hypothalamus [233,234] and is required for POMC neuron development [235], indicating a possible direct or indirect role for TBX3 in GnRH neuron function.

Recently, a chromosomal translocation t(3;13)(p13;q32) was reported to disrupt *ROBO1* (Roundabout Guidance Receptor 1), *ROBO2* and *SCEL* genes in a single patient with KS [236]. However, murine studies in compound *Robo1*/*Robo2* mutant mice did not reveal gross defects in the establishment of the GnRH neuronal system [237]. In the same study, mice lacking *Robo3* or its cognate receptor *Slit2* displayed slight defects in GnRH neuron migration, but these results have been recently disputed by Forni and colleagues, who did not detect any abnormalities [238]. Thus, it is plausible that a certain grade of redundancy exists in the Slit-Robo mouse system, and further studies in alternative models (e.g., human iPSCs) will be needed.

A *SEMA3G* point mutation was recently found by our group in two brothers affected by an unusual syndrome, comprising normosmic CHH, developmental delay and facial dysmorphic features. *Sema3g*-null mouse embryos displayed impaired GnRH neuron migration and it has been proposed that SEMA3G could partially hamper SEMA3A signaling by altering the binding affinity and selectivity to PLXNA co-receptors. However, adult mutant mice did not show any reproductive defects [239].

Variants in *DLG2*, which encodes a synaptic anchoring protein for NMDA receptors, have been found to segregate with delayed puberty and were additionally found in three patients presenting normosmic CHH. These variants were shown to downregulate *Gnrh1* expression in vitro and *Dlg2* expression was found in the MPOA of adult rats, suggesting a possible neuroendocrine role for DLG2 within the GnRH neuron system [240].

The *PTCH1* gene has been recently proposed as a CHH novel candidate gene [241], because four different variants were identified and predicted to be pathogenetic in a normosmic CHH/KS group of patients.

Notably, an increasing interest is emerging regarding the role of non-coding RNAs in regulating GnRH neuron development. For instance, the induced knockout of miRNA-processing enzyme *Dicer* in GnRH neurons resulted in hypogonadism and infertility in mice [242]. Similarly, the lack of specific miRNAs in mice also led to hypogonadism and infertility [242,243]. Consistent with these findings, a targeted Sanger sequencing screening of candidate miRNAs revealed a heterozygous variant in *MIR200A* in a KS patient, although the authors excluded it as the main cause of the KS phenotype [244]. In contrast, the balanced translocation t(7;12)(q22;q24), recently found in a unique KS patient, affects the function of the long non-coding RNA *RMST*, whose expression increases during neural crest cell differentiation, and alters the expression of several CHH-associated downstream genes [245]. Overall, it will be important to expand the screening of variants in non-coding RNAs in CHH patients and to perform experiments in animal models to functionally validate these variants.

## 5. Conclusions

CHH is a complex disease which presents a high degree of phenotypic manifestations, possibly due to the genetic variability, and the low penetrance and variable inheritance mode observed for some genes. Overall, due to these features, the identification of novel genes implicated in its pathogenesis remains a challenge. In addition, canonical genetic investigations in affected families are limited by the negative effect of hypogonadism on reproductive efficiency and, therefore, by the reduced number of patients. Finally, our biological knowledge on the HPG axis is still incomplete.

In recent years, to unveil new CHH causative genes, two main approaches have been adopted: i) screening of CHH patients with high throughput techniques and functional validation of the identified genes/variants by ad hoc in vitro or in vivo models; and ii) basic science-driven identification and validation of candidate genes involved in GnRH neuron physiology and the subsequent search for mutations in patients via interrogation of available cohorts of patients.

Through these approaches, a growing number of causative genes have been discovered. However, these account for approximately 50% of the overall CHH cases, leaving space for new mechanisms. The combination of state-of-the art gene expression techniques at single-cell resolution with cutting-edge sequencing technologies may boost and expedite the identification of the remaining genes.

## Figures and Tables

**Table 1:** List of known CHH-associated genes.

Gene	Function	Role in GnRH System	Mouse CHH-Related Phenotype	Human Phenotype	MIM Number
*AMH*	D/E	Migration and axon elongation	NA	nCHH/KS	261550
*AMHR2*	D/E	Migration and axon elongation	Abnormal nasal axon patterning; impaired GnRH neuron migration, reduced number of MPOA GnRH neuron; hypogonadism and subfertility	nCHH/KS	261550
*ANOS1*	D	Migration	NA	KS	308700
*AXL*	D	Survival	Increased apoptotic GnRH neurons, reduced number of MPOA GnRH neuron; delayed puberty	nCHH/KS	NA
*CCDC141*	D	Migration	Decreased GnRH neuron migration (nasal explants)	KS	NA
*CHD7*	D	Neurogenesis	OB hypoplasia, defective olfactory neuron neurogenesis, impaired senses of smell; defective GnRH neuron neurogenesis, reduced number of MPOA GnRH neuron; hypogonadism and delayed puberty	nCHH/KS + CHARGE	612370
*CHL1*	D	Migration and survival	Abnormal nasal axon patterning (hypothesized)	KS	NA
*DCC*	D/E	Migration and axon elongation	Abnormal nasal axon patterning; impaired GnRH neuron migration, reduced number of MPOA GnRH neuron	KS	NA
*DMXL2*	E	Signaling and secretion	Reduced number of MPOA GnRH neuron; hypogonadism, delayed puberty, subfertility	nCHH + PEPNS	616133
*DUSP6*	D/E	Neurogenesis and axon elongation (hypothesized)	NA	nCHH/KS	615269
*FEZF1*	D	Migration	OB hypoplasia, abnormal nasal axon patterning; impaired GnRH neuron migration	KS	616030
*FGF8*	D/E	Neurogenesis and axon elongation	Abnormal nasal axon patterning; absence of GnRH neurons	nCHH/KS + SOD	612702
*FGF17*	D/E	Neurogenesis and axon elongation (hypothesized)	NA	CHH + DWS	615270
*FGFR1*	D/E	Neurogenesis and axon elongation	Reduced number of nasal and MPOA GnRH neuron; delayed puberty, subfertility	nCHH/KS + HS and SHFM	147950
*FLRT3*	D/E	Neurogenesis and axon elongation (hypothesized)	NA	KS	615271
*GNRH1*	E	Signaling and secretion	Hypogonadism, infertility	nCHH	614841
*GLI3*	D	Migration	Impaired OECs formation, abnormal nasal axon patterning; impaired GnRH neuron migration, reduced number of MPOA GnRH neuron	KS + GCPS	175700
*HS6ST1*	D	Migration	Delayed puberty	nCHH/KS	614880
*IGSF10*	D	Migration	NA	nCHH	NA
*IL17RD*	D	Neurogenesis (hypothesized)	NA	KS	615267
*KISS1*	E	Signaling and secretion	Absent puberty, hypogonadism	nCHH	614842
*KISS1R*	E	Signaling and secretion	Absent puberty, hypogonadism	nCHH	614837
*KLB*	E	Signaling and secretion	Hypogonadism, delayed puberty, subfertility	nCHH	NA
*LEP*	E	Signaling and secretion	Hypogonadism, infertility	nCHH + obesity	614962
*LEPR*	E	Signaling and secretion	Infertility	nCHH + obesity	614963
*NDNF*	D	Migration	Abnormal nasal axon patterning; impaired GnRH neuron migration, reduced number of MPOA GnRH neuron	KS	618841
*NR0B1*	E	Signaling and secretion	Hypogonadism, infertility	nCHH + CAH	300200
*NRP1*	D	Migration	Abnormal nasal axon patterning; impaired GnRH neuron migration, reduced number of MPOA GnRH neuron	KS	NA
*NRP2*	D	Migration	Abnormal nasal axon patterning; impaired GnRH neuron migration, reduced number of MPOA GnRH neuron; hypogonadism	KS	NA
*NSMF*	D	Migration	Abnormal nasal axon patterning; impaired GnRH neuron migration (nasal explants), reduced number of MPOA GnRH neuron; hypogonadism, delayed puberty, subfertility	nCHH/KS	614838
*NTN1*	D/E	Migration and axon elongation	Abnormal nasal axon patterning; impaired GnRH neuron migration, reduced number of MPOA GnRH neuron	KS	618264
*OTUD4*	D	Uncertain	NA	nCHH + GHS	NA
*PCSK1*	E	Signaling and secretion	NA	nCHH + obesity	600955
*PLXNA1*	D	Migration	Abnormal nasal axon patterning; impaired GnRH neuron migration, reduced number of MPOA GnRH neuron	KS	NA
*PLXNA3*	D	Migration	Normal nasal axon patterning; normal GnRH neuron migration; normal gonadal size	nCHH/KS	NA
*PNPLA6*	E	Uncertain	NA	nCHH + GHS	215470
*POLR3A*	E	Uncertain	NA	nCHH + 4H	607694
*POLR3B*	E	Uncertain	NA	nCHH + 4H	614381
*PROK2*	D/E	Migration and axon elongation	OB hypoplasia; impaired GnRH neuron migration, reduced number of MPOA GnRH neuron; hypogonadism, absent puberty, infertility	nCHH/KS	610628
*PROKR2*	D/E	Migration and axon elongation	OB hypoplasia, abnormal nasal axon patterning; reduced number of MPOA GnRH neuron; hypogonadism	nCHH/KS + SOD	244200
*RNF216*	E	Uncertain	Hypogonadism, infertility	nCHH + GHS	212840
*SMCHD1*	D	Migration	NA	KS + BAMS	603457
*SEMA3A*	D	Migration	Abnormal nasal axon patterning; impaired GnRH neuron migration, reduced number of MPOA GnRH neuron; hypogonadism	nCHH/KS	614897
*SEMA3E*	D	Survival	Increased apoptotic GnRH neurons, reduced number of MPOA GnRH neuron; hypogonadism	KS + CHARGE?	214800
*SEMA3F*	D	Migration	Normal nasal axon patterning; normal GnRH neuron migration	nCHH/KS	NA
*SEMA7A*	D	Migration	Impaired GnRH neuron migration, reduced number of MPOA GnRH neuron; hypogonadism; hypogonadism, subfertility	nCHH/KS	NA
*SOX10*	D	Migration	Impaired OECs migration, abnormal nasal axon patterning; impaired GnRH neuron migration, reduced number of MPOA GnRH neuron	KS + WS and HD	602229
*SPRY4*	D/E	Migration and axon elongation (hypothesized)	NA	nCHH/KS	615266
*STUB1*	E	Uncertain	Hypogonadism	nCHH + GHS	NA
*TAC3*	E	Signaling and secretion	Delayed puberty	nCHH	614839
*TACR3*	E	Signaling and secretion	Hypogonadism	nCHH	618840
*TCF12*	D	Neurogenesis and migration	NA	KS + C	615314
*TUBB3*	D	Migration (hypothesized)	NA	KS + CFEOM	600638
*WDR11*	D	Neurogenesis	OB hypoplasia, reduced number of hypothalamic GnRH neurons; hypogonadism, delayed puberty, infertility	nCHH/KS + CPHD	614858

Abbreviations: D, neurodevelopmental; E, neuroendocrine; OB, olfactory bulb; MPOA, medial preoptic area; nCHH, normosmic congenital hypogonadotropic hypogonadism; KS, Kallmann syndrome; PEPNS, polyendocrine polyneuropathy syndrome; SOD, septo-optic dysplasia; HS, Hartsfield syndrome; DWS, Dandy–Walker syndrome; SHFM, split-hand/foot malformation; GCPS, Greig cephalopolysyndactyly; CAH, congenital adrenal hypoplasia; GHS, Gordon Holmes syndrome; 4H, hypomyelination, hypogonadotropic hypogonadism, and hypodontia; BAMS, Bosma arrhinia microphthalmia syndrome; WS, Waardenburg syndrome; HD, Hirschsprung disease; C, Craniosynostosis; CFEOM, Congenital fibrosis of the extraocular muscles; CPHD, combined pituitary hormone deficiency; NA, not available.

## References

[1] Kaprara A., Huhtaniemi I.T. (2018). The hypothalamus-pituitary-gonad axis: Tales of mice and men. Metabolism.

[2] Boehm U., Bouloux P.-M., Dattani M.T., de Roux N., Dodé C., Dunkel L., Dwyer A., Giacobini P., Hardelin J.-P., Juul A. (2015). Expert consensus document: European Consensus Statement on congenital hypogonadotropic hypogonadism—pathogenesis, diagnosis and treatment. Nat. Rev. Endocrinol..

[3] Casoni F., Malone S., Belle M., Luzzati F., Collier F., Allet C., Hrabovszky E., Rasika S., Prevot V., Chedotal A. (2016). Development of the neurons controlling fertility in humans: New insights from 3D imaging and transparent fetal brains. Development.

[4] Wray S. (2010). From Nose to Brain: Development of Gonadotrophin-Releasing Hormone -1 Neurones. J. Neuroendocr..

[5] Rance N.E., Young W.S., McMullen N.T. (1994). Topography of neurons expressing luteinizing hormone-releasing hormone gene transcripts in the human hypothalamus and basal forebrain. J. Comp. Neurol..

[6] Maier E.C., Saxena A., Alsina B., Bronner M.E., Whitfield T.T. (2014). Sensational placodes: Neurogenesis in the otic and olfactory systems. Dev. Biol..

[7] Forni P.E., Wray S. (2014). GnRH, anosmia and hypogonadotropic hypogonadism—Where are we?. Front. Neuroendocr..

[8] Forni P.E., Taylor-Burds C., Melvin V.S., Williams T., Wray S., Williams T. (2011). Neural Crest and Ectodermal Cells Intermix in the Nasal Placode to Give Rise to GnRH-1 Neurons, Sensory Neurons, and Olfactory Ensheathing Cells. J. Neurosci..

[9] Cho H.-J., Shan Y., Whittington N.C., Wray S. (2019). Nasal Placode Development, GnRH Neuronal Migration and Kallmann Syndrome. Front. Cell Dev. Biol..

[10] Taroc E.Z.M., Katreddi R.R., Forni P.E. (2020). Identifying Isl1 Genetic Lineage in the Developing Olfactory System and in GnRH-1 Neurons. Front. Physiol..

[11] Lund C., Yellapragada V., Vuoristo S., Balboa D., Trova S., Allet C., Eskici N., Pulli K., Giacobini P., Tuuri T. (2020). Characterization of the human GnRH neuron developmental transcriptome using a GNRH1-TdTomato reporter line in human pluripotent stem cells. Dis. Model. Mech..

[12] Cariboni A., Maggi R., Parnavelas J.G. (2007). From nose to fertility: The long migratory journey of gonadotropin-releasing hormone neurons. Trends Neurosci..

[13] Wierman M.E., Kiseljak-Vassiliades K., Tobet S. (2011). Gonadotropin-releasing hormone (GnRH) neuron migration: Initiation, maintenance and cessation as critical steps to ensure normal reproductive function. Front. Neuroendocr..

[14] Yoshida K., Tobet S.A., Crandall J.E., Jimenez T.P., Schwarting G.A. (1995). The migration of luteinizing hormone-releasing hormone neurons in the developing rat is associated with a transient, caudal projection of the vomeronasal nerve. J. Neurosci..

[15] Taroc E.Z., Prasad A., Lin J.M., Forni P.E. (2017). The terminal nerve plays a prominent role in GnRH-1 neuronal migration independent from proper olfactory and vomeronasal connections to the olfactory bulbs. Biol. Open.

[16] Messina A., Giacobini P. (2013). Semaphorin Signaling in the Development and Function of the Gonadotropin Hormone-Releasing Hormone System. Front. Endocrinol..

[17] Lettieri A., Oleari R., Gimmelli J., André V., Cariboni A. (2016). The role of semaphorin signaling in the etiology of hypogonadotropic hypogonadism. Minerva Endocrinol..

[18] Oleari R., Lettieri A., Paganoni A., Zanieri L., Cariboni A. (2018). Semaphorin Signaling in GnRH Neurons: From Development to Disease. Neuroendocrinology.

[19] Prevot V. (2015). Puberty in Mice and Rats. Knobil and Neill’s Physiology of Reproduction.

[20] Pierce A., Bliesner B., Xu M., Nielsen-Preiss S., Lemke G., Tobet S., Wierman M.E. (2008). Axl and Tyro3 Modulate Female Reproduction by Influencing Gonadotropin-Releasing Hormone Neuron Survival and Migration. Mol. Endocrinol..

[21] Muscatelli F., Abrous D.N., Massacrier A., Boccaccio I., Le Moal M., Cau P., Cremer H. (2000). Disruption of the mouse Necdin gene results in hypothalamic and behavioral alterations reminiscent of the human Prader-Willi syndrome. Hum. Mol. Genet..

[22] Cogliati T., Delgado-Romero P., Norwitz E.R., Guduric-Fuchs J., Kaiser U.B., Wray S., Kirsch I.R. (2007). Pubertal Impairment in Nhlh2 Null Mice Is Associated with Hypothalamic and Pituitary Deficiencies. Mol. Endocrinol..

[23] Cariboni A., André V., Chauvet S., Cassatella D., Davidson K., Caramello A., Fantin A., Bouloux P., Mann F., Ruhrberg C. (2015). Dysfunctional SEMA3E signaling underlies gonadotropin-releasing hormone neuron deficiency in Kallmann syndrome. J. Clin. Investig..

[24] Pitteloud N., Zhang C., Pignatelli D., Li J.-D., Raivio T., Cole L.W., Plummer L., Jacobson-Dickman E.E., Mellon P.L., Zhou Q.-Y. (2007). Loss-of-function mutation in the prokineticin 2 gene causes Kallmann syndrome and normosmic idiopathic hypogonadotropic hypogonadism. Proc. Natl. Acad. Sci. USA.

[25] Gill J.C., Tsai P.-S. (2006). Expression of a Dominant Negative FGF Receptor in Developing GNRH1 Neurons Disrupts Axon Outgrowth and Targeting to the Median Eminence. Biol. Reprod..

[26] Tsai P.-S., Moenter S.M., Postigo H.R., El Majdoubi M., Pak T.R., Gill J.C., Paruthiyil S., Werner S., Weiner R.I. (2005). Targeted Expression of a Dominant-Negative Fibroblast Growth Factor (FGF) Receptor in Gonadotropin-Releasing Hormone (GnRH) Neurons Reduces FGF Responsiveness and the Size of GnRH Neuronal Population. Mol. Endocrinol..

[27] Low V.F., Fiorini Z., Fisher L., Jasoni C.L. (2012). Netrin-1 Stimulates Developing GnRH Neurons to Extend Neurites to the Median Eminence in a Calcium- Dependent Manner. PLoS ONE.

[28] Parkash J., Cimino I., Ferraris N., Casoni F., Wray S., Cappy H., Prevot V., Giacobini P. (2012). Suppression of β1-integrin in gonadotropin-releasing hormone cells disrupts migration and axonal extension resulting in severe reproductive alterations. J. Neurosci..

[29] Herde M., Iremonger K., Constantin S., Herbison A.E. (2013). GnRH Neurons Elaborate a Long-Range Projection with Shared Axonal and Dendritic Functions. J. Neurosci..

[30] Moore A.M., Prescott M., Czieselsky K., Desroziers E., Yip S.H., Campbell R.E., Herbison A.E. (2018). Synaptic Innervation of the GnRH Neuron Distal Dendron in Female Mice. Endocrinology.

[31] Wang L., Guo W., Shen X., Yeo S., Long H., Wang Z., Lyu Q., Herbison A.E., Kuang Y. (2020). Different dendritic domains of the GnRH neuron underlie the pulse and surge modes of GnRH secretion in female mice. eLife.

[32] Clarke I., Arbabi L. (2016). New concepts of the central control of reproduction, integrating influence of stress, metabolic state, and season. Domest. Anim. Endocrinol..

[33] Herbison A.E. (2016). Control of puberty onset and fertility by gonadotropin-releasing hormone neurons. Nat. Rev. Endocrinol..

[34] Spergel D.J. (2018). Neuropeptidergic modulation of GnRH neuronal activity and GnRH secretion controlling reproduction: Insights from recent mouse studies. Cell Tissue Res..

[35] Cravo R., Margatho L., Osborne-Lawrence S., Donato J., Atkin S., Bookout A., Rovinsky S., Frazao R., Lee C., Gautron L. (2011). Characterization of Kiss1 neurons using transgenic mouse models. Neuroscience.

[36] Moore A.M., Coolen L.M., Porter D.T., Goodman R.L., Lehman M.N. (2018). KNDy Cells Revisited. Endocrinology.

[37] Navarro V.M., Gottsch M.L., Chavkin C., Okamura H., Clifton D.K., Steiner R.A. (2009). Regulation of Gonadotropin-Releasing Hormone Secretion by Kisspeptin/Dynorphin/Neurokinin B Neurons in the Arcuate Nucleus of the Mouse. J. Neurosci..

[38] Marino M., Moriondo V., Vighi E., Pignatti E., Simoni M. (2014). Central Hypogonadotropic Hypogonadism: Genetic Complexity of a Complex Disease. Int. J. Endocrinol..

[39] Stamou M.I., Georgopoulos N.A. (2017). Kallmann syndrome: Phenotype and genotype of hypogonadotropic hypogonadism. Metabolism.

[40] Young J., Xu C., Papadakis G.E., Acierno J.S., Maione L., Hietamäki J., Raivio T., Pitteloud N. (2019). Clinical Management of Congenital Hypogonadotropic Hypogonadism. Endocr. Rev..

[41] Cangiano B., Swee D.S., Quinton R., Bonomi M. (2020). Genetics of congenital hypogonadotropic hypogonadism: Peculiarities and phenotype of an oligogenic disease. Hum. Ganet..

[42] Renault C.H., Aksglaede L., Wøjdemann D., Hansen A.B., Jensen R.B., Juul A. (2020). Minipuberty of human infancy—A window of opportunity to evaluate hypogonadism and differences of sex development?. Ann. Pediatr. Endocrinol. Metab..

[43] Kuiri-Hänninen T., Sankilampi U., Dunkel L. (2014). Activation of the Hypothalamic-Pituitary-Gonadal Axis in Infancy: Minipuberty. Horm. Res. Paediatr..

[44] Xu C., Pitteloud N. (2019). Congenital Hypogonadotropic Hypogonadism (Isolated GnRH Deficiency). Contemporary Endocrinology.

[45] Varimo T., Hero M., Laitinen E.-M., Sintonen H., Raivio T. (2015). Health-related quality of life in male patients with congenital hypogonadotropic hypogonadism. Clin. Endocrinol..

[46] Laitinen E.-M., Hero M., Vaaralahti K., Tommiska J., Raivio T. (2012). Bone mineral density, body composition and bone turnover in patients with congenital hypogonadotropic hypogonadism. Int. J. Androl..

[47] Brand J.S., Rovers M., Yeap B.B., Schneider H.J., Tuomainen T.-P., Haring R., Corona G., Onat A., Maggio M., Bouchard C. (2014). Testosterone, Sex Hormone-Binding Globulin and the Metabolic Syndrome in Men: An Individual Participant Data Meta-Analysis of Observational Studies. PLoS ONE.

[48] Raivio T., Falardeau J., Dwyer A., Quinton R., Hayes F.J., Hughes V.A., Cole L.W., Pearce S., Lee H., Boepple P. (2007). Reversal of Idiopathic Hypogonadotropic Hypogonadism. N. Engl. J. Med..

[49] Sidhoum V.F., Chan Y.-M., Lippincott M., Balasubramanian R., Quinton R., Plummer L., Dwyer A., Pitteloud N., Hayes F.J., Hall J. (2014). Reversal and Relapse of Hypogonadotropic Hypogonadism: Resilience and Fragility of the Reproductive Neuroendocrine System. J. Clin. Endocrinol. Metab..

[50] Dodé C., Hardelin J.-P. (2009). Kallmann syndrome. Eur. J. Hum. Genet..

[51] Kim S.-H. (2015). Congenital Hypogonadotropic Hypogonadism and Kallmann Syndrome: Past, Present, and Future. Endocrinol. Metab..

[52] Nachtigall L.B., Boepple P.A., Pralong F.P., Crowley W.F. (1997). Adult-Onset Idiopathic Hypogonadotropic Hypogonadism—A Treatable Form of Male Infertility. N. Engl. J. Med..

[53] Tajar A., Forti G., O’Neill T.W., Lee D.M., Silman A.J., Finn J.D., Bartfai G., Boonen S., Casanueva F.F., Giwercman A. (2010). Characteristics of Secondary, Primary, and Compensated Hypogonadism in Aging Men: Evidence from the European Male Ageing Study. J. Clin. Endocrinol. Metab..

[54] Cangiano B., Duminuco P., Vezzoli V., Guizzardi F., Chiodini I., Corona G., Maggi M., Persani L., Bonomi M. (2019). Evidence for a Common Genetic Origin of Classic and Milder Adult-Onset Forms of Isolated Hypogonadotropic Hypogonadism. J. Clin. Med..

[55] Pitteloud N., Quinton R., Pearce S., Raivio T., Acierno J., Dwyer A., Plummer L., Hughes V., Seminara S., Cheng Y.-Z. (2007). Digenic mutations account for variable phenotypes in idiopathic hypogonadotropic hypogonadism. J. Clin. Investig..

[56] Sykiotis G., Plummer L., Hughes V.A., Au M., Durrani S., Nayak-Young S., Dwyer A., Quinton R., Hall J., Gusella J.F. (2010). Oligogenic basis of isolated gonadotropin-releasing hormone deficiency. Proc. Natl. Acad. Sci. USA.

[57] Cassatella D., Howard S., Acierno J., Xu C., Papadakis G.E., Santoni F., Dwyer A., Santini S., Sykiotis G., Chambion C. (2018). Congenital hypogonadotropic hypogonadism and constitutional delay of growth and puberty have distinct genetic architectures. Eur. J. Endocrinol..

[58] Maione L., Dwyer A., Francou B., Guiochon-Mantel A., Binart N., Bouligand J., Young J. (2018). Genetics in Endocrinology: Genetic counseling for congenital hypogonadotropic hypogonadism and Kallmann syndrome: New challenges in the era of oligogenism and next-generation sequencing. Eur. J. Endocrinol..

[59] Balasubramanian R., Crowley W.F. (1993). Isolated Gonadotropin-Releasing Hormone (GnRH) Deficiency. GeneReviews^®^.

[60] Constantin S. (2011). Physiology of the Gonadotrophin-Releasing Hormone (GnRH) Neurone: Studies from Embryonic GnRH Neurones. J. Neuroendocr..

[61] Palevitch O., Kight K., Abraham E., Wray S., Zohar Y., Gothilf Y. (2006). Ontogeny of the GnRH systems in zebrafish brain: In situ hybridization and promoter-reporter expression analyses in intact animals. Cell Tissue Res..

[62] Maggi R., Pimpinelli F., Molteni L., Milani M., Martini L., Piva F. (2000). Immortalized Luteinizing Hormone-Releasing Hormone Neurons Show a Different Migratory Activity in Vitro. Endocrinology.

[63] Louden E.D., Poch A., Kim H.-G., Ben-Mahmoud A., Kim S.-H., Layman L.C. (2021). Genetics of hypogonadotropic Hypogonadism—Human and mouse genes, inheritance, oligogenicity, and genetic counseling. Mol. Cell. Endocrinol..

[64] Mason A., Hayflick J., Zoeller R., Young W., Phillips H., Nikolics K., Seeburg P. (1986). A deletion truncating the gonadotropin-releasing hormone gene is responsible for hypogonadism in the hpg mouse. Science.

[65] Bouligand J., Ghervan C., Tello J.A., Brailly-Tabard S., Salenave S., Chanson P., Lombès M., Millar R.P., Guiochon-Mantel A., Young J. (2009). Isolated Familial Hypogonadotropic Hypogonadism and aGNRH1Mutation. N. Engl. J. Med..

[66] Chan Y.-M., de Guillebon A., Lang-Muritano M., Plummer L., Cerrato F., Tsiaras S., Gaspert A., Lavoie H.B., Wu C.-H., Crowley W.F. (2009). GNRH1mutations in patients with idiopathic hypogonadotropic hypogonadism. Proc. Natl. Acad. Sci. USA.

[67] Mengen E., Tunc S., Kotan L.D., Nalbantoglu O., Demir K., Gürbüz F., Turan I., Seker G., Yuksel B., Topaloglu A.K. (2015). Complete Idiopathic Hypogonadotropic Hypogonadism due to Homozygous GNRH1 Mutations in the Mutational Hot Spots in the Region Encoding the Decapeptide. Horm. Res. Paediatr..

[68] Horikoshi M., Day F.R., Akiyama M., Hirata M., Kamatani Y., Matsuda K., Ishigaki K., Kanai M., Wright H., Toro C.A. (2018). Elucidating the genetic architecture of reproductive ageing in the Japanese population. Nat. Commun..

[69] Messager S., Chatzidaki E.E., Ma D., Hendrick A., Zahn D., Dixon J., Thresher R.R., Malinge I., Lomet D., Carlton M.B.L. (2005). Kisspeptin directly stimulates gonadotropin-releasing hormone release via G protein-coupled receptor 54. Proc. Natl. Acad. Sci. USA.

[70] Topaloglu A.K., Tello J.A., Kotan L.D., Ozbek M.N., Yilmaz M.B., Erdogan S., Gürbüz F., Temiz F., Millar R.P., Yuksel B. (2012). InactivatingKISS1Mutation and Hypogonadotropic Hypogonadism. N. Engl. J. Med..

[71] De Roux N., Genin E., Carel J.-C., Matsuda F., Chaussain J.-L., Milgrom E. (2003). Hypogonadotropic hypogonadism due to loss of function of the KiSS1-derived peptide receptor GPR54. Proc. Natl. Acad. Sci. USA.

[72] Seminara S.B., Messager S., Chatzidaki E.E., Thresher R.R., Acierno J.S., Shagoury J.K., Bo-Abbas Y., Kuohung W., Schwinof K.M., Hendrick A. (2003). TheGPR54Gene as a Regulator of Puberty. N. Engl. J. Med..

[73] Colledge W. (2009). Transgenic mouse models to study Gpr54/kisspeptin physiology. Peptides.

[74] d’Anglemont de Tassigny X.D., Fagg L.A., Dixon J.P.C., Day K., Leitch H., Hendrick A., Zahn D., Franceschini I., Caraty A., Carlton M.B.L. (2007). Hypogonadotropic hypogonadism in mice lacking a functional Kiss1 gene. Proc. Natl. Acad. Sci. USA.

[75] Lapatto R., Pallais J.C., Zhang D., Chan Y.-M., Mahan A., Cerrato F., Le W.W., Hoffman G.E., Seminara S.B. (2007). Kiss1^−/−^ Mice Exhibit More Variable Hypogonadism than Gpr54^−/−^ Mice. Endocrinology.

[76] Chan Y.-M., Broder-Fingert S., Wong K.M., Seminara S.B. (2009). Kisspeptin/Gpr54-Independent Gonadotrophin-Releasing Hormone Activity inKiss1andGpr54Mutant Mice. J. Neuroendocr..

[77] Fujiyama T., Miyashita S., Tsuneoka Y., Kanemaru K., Kakizaki M., Kanno S., Ishikawa Y., Yamashita M., Owa T., Nagaoka M. (2018). Forebrain Ptf1a Is Required for Sexual Differentiation of the Brain. Cell Rep..

[78] Whittaker D.E., Oleari R., Gregory L., Le Quesne-Stabej P., Williams H., GOSgene, Torpiano J., Formosa N., Cachia M., Field D. (2021). A recessive PRDM13 mutation results in congenital hypogonadotropic hypogonadism and cerebellar hypoplasia. medRxiv.

[79] Hrabovszky E., Takács S., Göcz B., Skrapits K. (2019). New Perspectives for Anatomical and Molecular Studies of Kisspeptin Neurons in the Aging Human Brain. Neuroendocrinology.

[80] Clarke S.A., Dhillo W.S. (2016). Kisspeptin across the human lifespan: Evidence from animal studies and beyond. J. Endocrinol..

[81] Topaloglu A.K., Reimann F., Guclu M., Yalin A.S., Kotan L.D., Porter K.M., Serin A., Mungan N.O., Cook J., Imamoglu S. (2008). TAC3 and TACR3 mutations in familial hypogonadotropic hypogonadism reveal a key role for Neurokinin B in the central control of reproduction. Nat. Genet..

[82] Young J., Bouligand J., Francou B., Raffin-Sanson M.-L., Gaillez S., Jeanpierre M., Grynberg M., Kamenicky P., Chanson P., Brailly-Tabard S. (2010). TAC3andTACR3Defects Cause Hypothalamic Congenital Hypogonadotropic Hypogonadism in Humans. J. Clin. Endocrinol. Metab..

[83] Gianetti E., Tusset C., Noel S.D., Au M.G., Dwyer A., Hughes V.A., Abreu A.P., Carroll J., Trarbach E., Silveira L. (2010). TAC3/TACR3 Mutations Reveal Preferential Activation of Gonadotropin-Releasing Hormone Release by Neurokinin B in Neonatal Life Followed by Reversal in Adulthood. J. Clin. Endocrinol. Metab..

[84] True C., Alam S.N., Cox K., Chan Y.-M., Seminara S.B. (2015). Neurokinin B Is Critical for Normal Timing of Sexual Maturation but Dispensable for Adult Reproductive Function in Female Mice. Endocrinology.

[85] Yang J.J., Caligioni C.S., Chan Y.-M., Seminara S.B. (2012). Uncovering Novel Reproductive Defects in Neurokinin B Receptor Null Mice: Closing the Gap Between Mice and Men. Endocrinology.

[86] Strobel A., Issad T., Camoin L., Ozata M., Strosberg A.D. (1998). A leptin missense mutation associated with hypogonadism and morbid obesity. Nat. Genet..

[87] Clément K., Vaisse C., Lahlou N., Cabrol S., Pelloux V., Cassuto D., Gourmelen M., Dina C., Chambaz J., Lacorte J.-M. (1998). A mutation in the human leptin receptor gene causes obesity and pituitary dysfunction. Nature.

[88] Manfredi-Lozano M., Roa J., Tena-Sempere M. (2018). Connecting metabolism and gonadal function: Novel central neuropeptide pathways involved in the metabolic control of puberty and fertility. Front. Neuroendocr..

[89] Quennell J.H., Mulligan A.C., Tups A., Liu X., Phipps S.J., Kemp C.J., Herbison A., Grattan D., Anderson G.M. (2009). Leptin Indirectly Regulates Gonadotropin-Releasing Hormone Neuronal Function. Endocrinology.

[90] Landry D., Cloutier F., Martin L.J. (2013). Implications of leptin in neuroendocrine regulation of male reproduction. Reprod. Biol..

[91] Zhang Y., Proenca R., Maffei M., Barone M., Leopold L., Friedman J.M. (1994). Positional cloning of the mouse obese gene and its human homologue. Nature.

[92] Cohen P., Zhao C., Cai X., Montez J.M., Rohani S.C., Feinstein P., Mombaerts P., Friedman J.M. (2001). Selective deletion of leptin receptor in neurons leads to obesity. J. Clin. Investig..

[93] Stijnen P., Ramos-Molina B., O’Rahilly S., Creemers J.W.M. (2016). PCSK1 Mutations and Human Endocrinopathies: From Obesity to Gastrointestinal Disorders. Endocr. Rev..

[94] O’Rahilly S., Gray H., Humphreys P.J., Krook A., Polonsky K.S., White A., Gibson S., Taylor K., Carr C. (1995). Brief Report: Impaired Processing of Prohormones Associated with Abnormalities of Glucose Homeostasis and Adrenal Function. N. Engl. J. Med..

[95] Jackson R.S., Creemers J.W.M., Ohagi S., Raffin-Sanson M.-L., Sanders L., Montague C., Hutton J.C., O’Rahilly S. (1997). Obesity and impaired prohormone processing associated with mutations in the human prohormone convertase 1 gene. Nat. Genet..

[96] Farooqi S., O’Rahilly S. (2006). Genetics of Obesity in Humans. Endocr. Rev..

[97] Farooqi I.S., Volders K., Stanhope R., Heuschkel R., White A., Lank E., Keogh J., O’Rahilly S., Creemers J.W.M. (2007). Hyperphagia and Early-Onset Obesity due to a Novel Homozygous Missense Mutation in Prohormone Convertase 1/3. J. Clin. Endocrinol. Metab..

[98] Sun H.D., Malabunga M., Tonra J.R., DiRenzo R., Carrick F.E., Zheng H., Berthoud H.-R., McGuinness O.P., Shen J., Bohlen P. (2007). Monoclonal antibody antagonists of hypothalamic FGFR1 cause potent but reversible hypophagia and weight loss in rodents and monkeys. Am. J. Physiol. Metab..

[99] Tacer K.F., Bookout A.L., Ding X., Kurosu H., John G.B., Wang L., Goetz R., Mohammadi M., Kuro-O M., Mangelsdorf D. (2010). Research Resource: Comprehensive Expression Atlas of the Fibroblast Growth Factor System in Adult Mouse. Mol. Endocrinol..

[100] Xu C., Messina A., Somm E., Miraoui H., Kinnunen T.K., Acierno J., Niederländer N.J., Bouilly J., Dwyer A., Sidis Y. (2017). KLB, encoding β-Klotho, is mutated in patients with congenital hypogonadotropic hypogonadism. EMBO Mol. Med..

[101] Wang Y., Sun Z. (2009). Current understanding of klotho. Ageing Res. Rev..

[102] Kuro-O M., Matsumura Y., Aizawa H., Kawaguchi H., Suga T., Utsugi T., Ohyama Y., Kurabayashi M., Kaname T., Kume E. (1997). Mutation of the mouse klotho gene leads to a syndrome resembling ageing. Nature.

[103] Wu S.-M., Gao J.-Z., He B., Long W.-J., Luo X.-P., Chen L. (2020). A Novel NR0B1 Gene Mutation Causes Different Phenotypes in Two Male Patients with Congenital Adrenal Hypoplasia. Curr. Med. Sci..

[104] Reutens A.T., Achermann J.C., Ito M., Ito M., Gu W.-X., Habiby R.L., Donohoue P.A., Pang S., Hindmarsh P.C., Jameson J.L. (1999). Clinical and Functional Effects of Mutations in theDAX-1Gene in Patients with Adrenal Hypoplasia Congenita. J. Clin. Endocrinol. Metab..

[105] Iyer A.K., McCabe E.R. (2004). Molecular mechanisms of DAX1 action. Mol. Genet. Metab..

[106] Li N., Liu R., Zhang H., Yang J., Sun S., Zhang M., Liu Y., Lu Y., Wang W., Mu Y. (2010). Seven Novel DAX1 Mutations with Loss of Function Identified in Chinese Patients with Congenital Adrenal Hypoplasia. J. Clin. Endocrinol. Metab..

[107] Achermann J., Meeks J.J., Jameson J.L. (2001). Phenotypic spectrum of mutations in DAX-1 and SF-1. Mol. Cell. Endocrinol..

[108] Muscatelli F., Strom T.M., Walker A.P., Zanaria E., Récan D., Meindl A., Bardoni B., Guioli S., Zehetner G., Rabl W. (1994). Mutations in the DAX-1 gene give rise to both X-linked adrenal hypoplasia congenita and hypogonadotropic hypogonadism. Nature.

[109] Seminara S.B., Achermann J.C., Genel M., Jameson J.L., Crowley W.F. (1999). X-Linked Adrenal Hypoplasia Congenita: A Mutation in DAX1 Expands the Phenotypic Spectrum in Males and Females. J. Clin. Endocrinol. Metab..

[110] Tata B., Huijbregts L., Jacquier S., Csaba Z., Genin E., Meyer V., Leka S., Dupont J., Charles P., Chevenne D. (2014). Haploinsufficiency of Dmxl2, Encoding a Synaptic Protein, Causes Infertility Associated with a Loss of GnRH Neurons in Mouse. PLoS Biol..

[111] Tata B.K., Harbulot C., Csaba Z., Peineau S., Jacquier S., De Roux N. (2017). Rabconnectin-3α is required for the morphological maturation of GnRH neurons and kisspeptin responsiveness. Sci. Rep..

[112] Winrow C.J., Hemming M.L., Allen D.M., Quistad G.B., Casida J.E., Barlow C. (2003). Loss of neuropathy target esterase in mice links organophosphate exposure to hyperactivity. Nat. Genet..

[113] Topaloglu A.K., Lomniczi A., Kretzschmar D., Dissen G.A., Kotan L.D., McArdle C.A., Koc A.F., Hamel B.C., Guclu M., Papatya E.D. (2014). Loss-of-Function Mutations inPNPLA6Encoding Neuropathy Target Esterase Underlie Pubertal Failure and Neurological Deficits in Gordon Holmes Syndrome. J. Clin. Endocrinol. Metab..

[114] Margolin D.H., Kousi M., Chan Y.-M., Lim T.T., Schmahmann J.D., Hadjivassiliou M., Hall J., Adam I., Dwyer A., Plummer L. (2013). Ataxia, Dementia, and Hypogonadotropism Caused by Disordered Ubiquitination. N. Engl. J. Med..

[115] Shi C.-H., Schisler J., Rubel C.E., Tan S., Song B., McDonough H., Xu L., Portbury A.L., Mao C.-Y., True C. (2013). Ataxia and hypogonadism caused by the loss of ubiquitin ligase activity of the U box protein CHIP. Hum. Mol. Genet..

[116] Melnick A., Gao Y., Liu J., Ding D., Predom A., Kelly C., Hess R.A., Chen C. (2019). RNF216 is essential for spermatogenesis and male fertility. Biol. Reprod..

[117] Li F., Li D., Liu H., Cao B.-B., Jiang F., Chen D.-N., Li J.-D. (2019). RNF216 Regulates the Migration of Immortalized GnRH Neurons by Suppressing Beclin1-Mediated Autophagy. Front. Endocrinol..

[118] Dumay-Odelot H., Durrieu-Gaillard S., Da Silva D., Roeder R.G., Teichmann M. (2010). Cell growth- and differentiation-dependent regulation of RNA polymerase III transcription. Cell Cycle.

[119] Wolff A., Koch M.J., Benzinger S., Van Waes H., Wolf N.I., Boltshauser E., Luder H.U. (2010). Rare dental peculiarities associated with the hypomyelinating leukoencephalopathy 4H syndrome/ADDH. Pediatr. Dent..

[120] Daoud H., Tétreault M., Gibson W., Guerrero K., Cohen A., Gburek-Augustat J., Synofzik M., Brais B., Stevens C.A., Sanchez-Carpintero R. (2013). Mutations inPOLR3AandPOLR3Bare a major cause of hypomyelinating leukodystrophies with or without dental abnormalities and/or hypogonadotropic hypogonadism. J. Med. Genet..

[121] Choquet K., Yang S., Moir R.D., Forget D., Larivière R., Bouchard A., Poitras C., Sgarioto N., Dicaire M.-J., Noohi F. (2017). Absence of neurological abnormalities in mice homozygous for the Polr3a G672E hypomyelinating leukodystrophy mutation. Mol. Brain.

[122] Choquet K., Pinard M., Yang S., Moir R.D., Poitras C., Dicaire M.-J., Sgarioto N., Larivière R., Kleinman C.L., Willis I.M. (2019). The leukodystrophy mutation Polr3b R103H causes homozygote mouse embryonic lethality and impairs RNA polymerase III biogenesis. Mol. Brain.

[123] Franco B., Guioli S., Pragliola A., Incerti B., Bardoni B., Tonlorenzi R., Carrozzo R., Maestrini E., Pieretti M., Taillon-Miller P. (1991). A gene deleted in Kallmann’s syndrome shares homology with neural cell adhesion and axonal path-finding molecules. Nature.

[124] Schwanzel-Fukuda M., Bick D., Pfaff D.W. (1989). Luteinizing hormone-releasing hormone (LHRH)-expressing cells do not migrate normally in an inherited hypogonadal (Kallmann) syndrome. Brain Res. Mol. Brain Res..

[125] Bulow H.E., Berry K., Topper L.H., Peles E., Hobert O. (2002). Heparan sulfate proteoglycan-dependent induction of axon branching and axon misrouting by the Kallmann syndrome gene kal-1. Proc. Natl. Acad. Sci. USA.

[126] Soussi-Yanicostas N., de Castro F., Julliard A.K., Perfettini I., Chedotal A., Petit C. (2002). Anosmin-1, Defective in the X-Linked Form of Kallmann Syndrome, Promotes Axonal Branch Formation from Olfactory Bulb Output Neurons. Cell.

[127] Cariboni A., Pimpinelli F., Colamarino S., Zaninetti R., Piccolella M., Rumio C., Piva F., Rugarli E.I., Maggi R. (2004). The product of X-linked Kallmann’s syndrome gene (KAL1) affects the migratory activity of gonadotropin-releasing hormone (GnRH)-producing neurons. Hum. Mol. Genet..

[128] Azzarelli R., Oleari R., Lettieri A., Andre’ V., Cariboni A. (2017). In Vitro, Ex Vivo and In Vivo Techniques to Study Neuronal Migration in the Developing Cerebral Cortex. Brain Sci..

[129] Costa-Barbosa F.A., Balasubramanian R., Keefe K.W., Shaw N., Al Tassan N., Plummer L., Dwyer A., Buck C.L., Choi J.-H., Seminara S.B. (2013). Prioritizing Genetic Testing in Patients With Kallmann Syndrome Using Clinical Phenotypes. J. Clin. Endocrinol. Metab..

[130] Layman L.C. (1999). Mutations in human gonadotropin genes and their physiologic significance in puberty and reproduction. Fertil. Steril..

[131] Tukiainen T., Villani A.-C., Yen A., Rivas M.A., Marshall J.L., Satija R., Aguirre M., Gauthier L., Fleharty M., GTEx Consortium (2017). Landscape of X chromosome inactivation across human tissues. Nature.

[132] Dodé C., Levilliers J., Dupont J.-M., De Paepe A., Le Dû N., Soussi-Yanicostas N., Coimbra R., Delmaghani S., Compain-Nouaille S., Baverel F. (2003). Loss-of-function mutations in FGFR1 cause autosomal dominant Kallmann syndrome. Nat. Genet..

[133] Tornberg J., Sykiotis G., Keefe K., Plummer L., Hoang X., Hall J., Quinton R., Seminara S.B., Hughes V., Van Vliet G. (2011). Heparan sulfate 6-O-sulfotransferase 1, a gene involved in extracellular sugar modifications, is mutated in patients with idiopathic hypogonadotrophic hypogonadism. Proc. Natl. Acad. Sci. USA.

[134] Condomitti G., De Wit J. (2018). Heparan Sulfate Proteoglycans as Emerging Players in Synaptic Specificity. Front. Mol. Neurosci..

[135] Sarrazin S., Lamanna W.C., Esko J.D. (2011). Heparan Sulfate Proteoglycans. Cold Spring Harb. Perspect. Biol..

[136] Habuchi H., Nagai N., Sugaya N., Atsumi F., Stevens R.L., Kimata K. (2007). Mice Deficient in Heparan Sulfate 6-O-Sulfotransferase-1 Exhibit Defective Heparan Sulfate Biosynthesis, Abnormal Placentation, and Late Embryonic Lethality. J. Biol. Chem..

[137] Howard S., Oleari R., Poliandri A., Chantzara V., Fantin A., Ruiz-Babot G., Metherell L.A., Cabrera C.P., Barnes M.R., Wehkalampi K. (2018). HS6ST1 Insufficiency Causes Self-Limited Delayed Puberty in Contrast with Other GnRH Deficiency Genes. J. Clin. Endocrinol. Metab..

[138] Kramer P., Wray S. (2000). Novel gene expressed in nasal region influences outgrowth of olfactory axons and migration of luteinizing hormone-releasing hormone (LHRH) neurons. Genes Dev..

[139] Palevitch O., Abraham E., Borodovsky N., Levkowitz G., Zohar Y., Gothilf Y. (2009). Nasal embryonic LHRH factor plays a role in the developmental migration and projection of gonadotropin-releasing hormone 3 neurons in zebrafish. Dev. Dyn..

[140] Quaynor S.D., Ko E.K., Chorich L.P., Sullivan M.E., Demir D., Waller J.L., Kim H.-G., Cameron R.S., Layman L.C. (2015). NELF knockout is associated with impaired pubertal development and subfertility. Mol. Cell. Endocrinol..

[141] Miura K., Acierno J., Seminara S.B. (2004). Characterization of the human nasal embryonic LHRH factor gene, NELF, and a mutation screening among 65 patients with idiopathic hypogonadotropic hypogonadism (IHH). J. Hum. Genet..

[142] Lemke G. (2013). Biology of the TAM Receptors. Cold Spring Harb. Perspect. Biol..

[143] Salian-Mehta S., Xu M., Knox A.J., Plummer L., Slavov D., Taylor M., Bevers S., Hodges R.S., Crowley W.F., Wierman M.E. (2014). Functional consequences of AXL sequence variants in hypogonadotropic hypogonadism. J. Clin. Endocrinol. Metab..

[144] Eckler M.J., Chen B. (2014). Fez family transcription factors: Controlling neurogenesis and cell fate in the developing mammalian nervous system. BioEssays.

[145] Kotan L.D., Hutchins B.I., Ozkan Y., Demirel F., Stoner H., Cheng P.J., Esen I., Gürbüz F., Bicakci Y.K., Mengen E. (2014). Mutations in FEZF1 Cause Kallmann Syndrome. Am. J. Hum. Genet..

[146] Hutchins B.I., Kotan L.D., Taylor-Burds C., Ozkan Y., Cheng P.J., Gürbüz F., Tiong J.D.R., Mengen E., Yuksel B., Topaloglu A.K. (2016). CCDC141 Mutation Identified in Anosmic Hypogonadotropic Hypogonadism (Kallmann Syndrome) Alters GnRH Neuronal Migration. Endocrinology.

[147] Turan I., Hutchins B.I., Hacihamdioglu B., Kotan L.D., Gurbuz F., Ulubay A., Mengen E., Yuksel B., Wray S., Topaloglu A.K. (2017). CCDC141 Mutations in Idiopathic Hypogonadotropic Hypogonadism. J. Clin. Endocrinol. Metab..

[148] Watanabe Y., Inoue K., Okuyama-Yamamoto A., Nakai N., Nakatani J., Nibu K.-I., Sato N., Iiboshi Y., Yusa K., Kondoh G. (2009). Fezf1is required for penetration of the basal lamina by olfactory axons to promote olfactory development. J. Comp. Neurol..

[149] Kuang X.-L., Zhao X.-M., Xu H.-F., Shi Y.-Y., Deng J.-B., Sun G.-T. (2010). Spatio-temporal expression of a novel neuron-derived neurotrophic factor (NDNF) in mouse brains during development. BMC Neurosci..

[150] Messina A., Pulli K., Santini S., Acierno J., Känsäkoski J., Cassatella D., Xu C., Casoni F., Malone S.A., Ternier G. (2019). Neuron-Derived Neurotrophic Factor Is Mutated in Congenital Hypogonadotropic Hypogonadism. Am. J. Hum. Genet..

[151] Tamaoka S., Suzuki E., Hattori A., Ogata T., Fukami M., Katoh-Fukui Y. (2021). NDNF variants are rare in patients with congenital hypogonadotropic hypogonadism. Hum. Genome Var..

[152] Kim H.-G., Ahn J.-W., Kurth I., Ullmann R., Kim H.-T., Kulharya A., Ha K.-S., Itokawa Y., Meliciani I., Wenzel W. (2010). WDR11, a WD Protein that Interacts with Transcription Factor EMX1, Is Mutated in Idiopathic Hypogonadotropic Hypogonadism and Kallmann Syndrome. Am. J. Hum. Genet..

[153] Kim Y., Osborn D., Lee J., Araki M., Araki K., Mohun T., Känsäkoski J., Brandstack N., Kim H., Miralles F. (2017). WDR11-mediated Hedgehog signalling defects underlie a new ciliopathy related to Kallmann syndrome. EMBO Rep..

[154] McCormack S.E., Li N., Kim Y.J., Lee J.Y., Kim S.-H., Rapaport R., Levine M. (2017). Digenic Inheritance of PROKR2 and WDR11 Mutations in Pituitary Stalk Interruption Syndrome. J. Clin. Endocrinol. Metab..

[155] Cariboni A., Davidson K., Rakic S., Maggi R., Parnavelas J.G., Ruhrberg C. (2010). Defective gonadotropin-releasing hormone neuron migration in mice lacking SEMA3A signalling through NRP1 and NRP2: Implications for the aetiology of hypogonadotropic hypogonadism. Hum. Mol. Genet..

[156] Alto L.T., Terman J.R. (2016). Semaphorins and their Signaling Mechanisms. Methods Mol. Biol..

[157] Cariboni A., Hickok J., Rakic S., Andrews W., Maggi R., Tischkau S., Parnavelas J.G. (2007). Neuropilins and Their Ligands Are Important in the Migration of Gonadotropin-Releasing Hormone Neurons. J. Neurosci..

[158] Hanchate N.K., Giacobini P., Lhuillier P., Parkash J., Espy C., Fouveaut C., Leroy C., Baron S., Campagne C., Vanacker C. (2012). SEMA3A, a Gene Involved in Axonal Pathfinding, Is Mutated in Patients with Kallmann Syndrome. PLoS Genet..

[159] Oleari R., Caramello A., Campinoti S., Lettieri A., Ioannou E., Paganoni A., Fantin A., Cariboni A., Ruhrberg C. (2019). PLXNA1 and PLXNA3 cooperate to pattern the nasal axons that guide gonadotropin-releasing hormone neurons. Development.

[160] Marcos S., Monnier C., Rovira X., Fouveaut C., Pitteloud N., Ango F., Dodé C., Hardelin J.-P. (2017). Defective signaling through plexin-A1 compromises the development of the peripheral olfactory system and neuroendocrine reproductive axis in mice. Hum. Mol. Genet..

[161] Young J., Metay C., Bouligand J., Tou B., Francou B., Maione L., Tosca L., Sarfati J., Brioude F., Esteva B. (2012). SEMA3A deletion in a family with Kallmann syndrome validates the role of semaphorin 3A in human puberty and olfactory system development. Hum. Reprod..

[162] Känsäkoski J., Fagerholm R., Laitinen E.-M., Vaaralahti K., Hackman P., Pitteloud N., Raivio T., Tommiska J. (2014). Mutation screening of SEMA3A and SEMA7A in patients with congenital hypogonadotropic hypogonadism. Pediatr. Res..

[163] Kotan L.D., Isik E., Turan I., Mengen E., Akkus G., Tastan M., Gurbuz F., Yuksel B., Topaloglu A.K. (2018). Prevalence and associated phenotypes of PLXNA1 variants in normosmic and anosmic idiopathic hypogonadotropic hypogonadism. Clin. Genet..

[164] Kotan L.D., Ternier G., Cakir A.D., Emeksiz H.C., Turan I., Delpouve G., Kardelen A.D., Ozcabi B., Isik E., Mengen E. (2021). Loss-of-function variants in SEMA3F and PLXNA3 encoding semaphorin-3F and its receptor plexin-A3 respectively cause idiopathic hypogonadotropic hypogonadism. Genet. Med..

[165] Takeuchi H., Inokuchi K., Aoki M., Suto F., Tsuboi A., Matsuda I., Suzuki M., Aiba A., Serizawa S., Yoshihara Y. (2010). Sequential Arrival and Graded Secretion of Sema3F by Olfactory Neuron Axons Specify Map Topography at the Bulb. Cell.

[166] Chen Y., Sun T., Niu Y., Wang D., Liu K., Wang T., Wang S., Xu H., Liu J. (2021). Cell adhesion molecule L1 like plays a role in the pathogenesis of idiopathic hypogonadotropic hypogonadism. J. Endocrinol. Investig..

[167] Heyden A., Angenstein F., Sallaz M., Seidenbecher C., Montag D. (2008). Abnormal axonal guidance and brain anatomy in mouse mutants for the cell recognition molecules close homolog of L1 and NgCAM-related cell adhesion molecule. Neuroscience.

[168] Chauvet S., Cohen S., Yoshida Y., Fekrane L., Livet J., Gayet O., Segu L., Buhot M.-C., Jessell T.M., Henderson C. (2007). Gating of Sema3E/PlexinD1 Signaling by Neuropilin-1 Switches Axonal Repulsion to Attraction during Brain Development. Neuron.

[169] Bellon A., Luchino J., Haigh K., Rougon G., Haigh J., Chauvet S., Mann F. (2010). VEGFR2 (KDR/Flk1) Signaling Mediates Axon Growth in Response to Semaphorin 3E in the Developing Brain. Neuron.

[170] Lalani S.R., Safiullah A.M., Molinari L.M., Fernbach S.D., Martin D.M., Belmont J.W. (2004). SEMA3E mutation in a patient with CHARGE syndrome. J. Med. Genet..

[171] Lettieri A., Oleari R., Paganoni A.J.J., Gervasini C., Massa V., Fantin A., Cariboni A. (2021). Semaphorin Regulation by the Chromatin Remodeler CHD7: An Emerging Genetic Interaction Shaping Neural Cells and Neural Crest in Development and Cancer. Front. Cell Dev. Biol..

[172] Messina A., Ferraris N., Wray S., Cagnoni G., Donohue D., Casoni F., Kramer P., Derijck A.A., Adolfs Y., Fasolo A. (2011). Dysregulation of Semaphorin7A/β1-integrin signaling leads to defective GnRH-1 cell migration, abnormal gonadal development and altered fertility. Hum. Mol. Genet..

[173] Howard S., Guasti L., Ruiz-Babot G., Mancini A., David A., Storr H., Metherell L., Sternberg M., Cabrera C.P., Warren H.R. (2016). IGSF 10 mutations dysregulate gonadotropin-releasing hormone neuronal migration resulting in delayed puberty. EMBO Mol. Med..

[174] Sanlaville D., Etchevers H., Gonzales M., Martinovic J., Clément-Ziza M., Delezoide A.-L., Aubry M.-C., Pelet A., Chemouny S., Cruaud C. (2005). Phenotypic spectrum of CHARGE syndrome in fetuses with CHD7 truncating mutations correlates with expression during human development. J. Med. Genet..

[175] Layman W., McEwen D., Beyer L., Lalani S., Fernbach S., Oh E., Swaroop A., Hegg C., Raphael Y., Martens J. (2009). Defects in neural stem cell proliferation and olfaction in Chd7 deficient mice indicate a mechanism for hyposmia in human CHARGE syndrome. Hum. Mol. Genet..

[176] Bergman J.E.H., Bosman E.A., Van Ravenswaaij-Arts C.M., Steel K.P. (2009). Study of smell and reproductive organs in a mouse model for CHARGE syndrome. Eur. J. Hum. Genet..

[177] Layman W.S., Hurd E.A., Martin D.M. (2011). Reproductive dysfunction and decreased GnRH neurogenesis in a mouse model of CHARGE syndrome. Hum. Mol. Genet..

[178] Vissers L., Van Ravenswaaij C.M.A., Admiraal R., Hurst J.A., De Vries B.B.A., Janssen I.M., Van Der Vliet W.A., Huys E.H.L.P.G., De Jong P.J., Hamel B.C.J. (2004). Mutations in a new member of the chromodomain gene family cause CHARGE syndrome. Nat. Genet..

[179] Teixeira L., Guimiot F., Dodé C., Fallet-Bianco C., Millar R.P., Delezoide A.-L., Hardelin J.-P. (2010). Defective migration of neuroendocrine GnRH cells in human arrhinencephalic conditions. J. Clin. Investig..

[180] Kim H.-G., Kurth I., Lan F., Meliciani I., Wenzel W., Eom S.H., Kang G.B., Rosenberger G., Tekin M., Ozata M. (2008). Mutations in CHD7, Encoding a Chromatin-Remodeling Protein, Cause Idiopathic Hypogonadotropic Hypogonadism and Kallmann Syndrome. Am. J. Hum. Genet..

[181] Jongmans M.C.J., Van Ravenswaaij-Arts C.M.A., Pitteloud N., Ogata T., Sato N., Claahsen-van der Grinten H.L., Van Der Donk K., Seminara S., Bergman J., Brunner H.G. (2008). CHD7mutations in patients initially diagnosed with Kallmann syndrome—The clinical overlap with CHARGE syndrome. Clin. Genet..

[182] Xu C., Cassatella D., Van Der Sloot A.M., Quinton R., Hauschild M., De Geyter C., Flueck C., Feller K., Bartholdi D., Nemeth A. (2017). Evaluating CHARGE syndrome in congenital hypogonadotropic hypogonadism patients harboring CHD7 variants. Genet. Med..

[183] Schulz Y., Wehner P., Opitz L., Salinas-Riester G., Bongers E.M.H.F., Van Ravenswaaij-Arts C.M.A., Wincent J., Schoumans J., Kohlhase J., Borchers A. (2014). CHD7, the gene mutated in CHARGE syndrome, regulates genes involved in neural crest cell guidance. Hum. Ganet..

[184] Ufartes R., Schwenty-Lara J., Freese L., Neuhofer C.M., Möller J., Wehner P., Van Ravenswaaij-Arts C.M.A., Wong M.T.Y., Schanze I., Tzschach A. (2018). Sema3a plays a role in the pathogenesis of CHARGE syndrome. Hum. Mol. Genet..

[185] Barraud P., John J.S., Stolt C.C., Wegner M., Baker C.V.H. (2013). Olfactory ensheathing glia are required for embryonic olfactory axon targeting and the migration of gonadotropin-releasing hormone neurons. Biol. Open.

[186] Pingault V., Bodereau V., Baral V., Marcos S., Watanabe Y., Chaoui A., Fouveaut C., Leroy C., Vérier-Mine O., Francannet C. (2013). Loss-of-Function Mutations in SOX10 Cause Kallmann Syndrome with Deafness. Am. J. Hum. Genet..

[187] Inoue K., Khajavi M., Ohyama T., Hirabayashi S.-I., Wilson J.H., Reggin J.D., Mancias P., Butler I.J., Wilkinson M.F., Wegner M. (2004). Molecular mechanism for distinct neurological phenotypes conveyed by allelic truncating mutations. Nat. Genet..

[188] Rojas R.A., Kutateladze A.A., Plummer L., Stamou M., Keefe D.L.K., Salnikov K.B., Delaney A., Hall J.E., Sadreyev R., Ji F. (2021). Phenotypic continuum between Waardenburg syndrome and idiopathic hypogonadotropic hypogonadism in humans with SOX10 variants. Genet. Med..

[189] Blewitt M., Gendrel A.-V., Pang Z., Sparrow D.B., Whitelaw N., Craig J.M., Apedaile A., Hilton D.J., Dunwoodie S.L., Brockdorff N. (2008). SmcHD1, containing a structural-maintenance-of-chromosomes hinge domain, has a critical role in X inactivation. Nat. Genet..

[190] Shaw N., Brand H., Kupchinsky Z.A., Bengani H., Plummer L., Jones T.I., Erdin S., Williamson K.A., Rainger J., Stortchevoi A. (2017). SMCHD1 mutations associated with a rare muscular dystrophy can also cause isolated arhinia and Bosma arhinia microphthalmia syndrome. Nat. Genet..

[191] Gordon C.T., Xue S., Yigit G., Filali H., Chen K., Rosin N., Yoshiura K.-I., Oufadem M., Beck T.J., McGowan R. (2017). De novo mutations in SMCHD1 cause Bosma arhinia microphthalmia syndrome and abrogate nasal development. Nat. Genet..

[192] Delaney A., Volochayev R., Meader B., Lee J., Almpani K., Noukelak G.Y., Henkind J., Chalmers L., Law J.R., Williamson K.A. (2020). Insight Into the Ontogeny of GnRH Neurons From Patients Born Without a Nose. J. Clin. Endocrinol. Metab..

[193] Sharma V.P., Mulliken J.B., Fenwick A.L., Brockop M.S., McGowan S., Goos J.A.C., Hoogeboom A.J.M., Brady A.F., Jeelani N.U.O., Lynch S.A. (2013). Mutations in TCF12, encoding a basic helix-loop-helix partner of TWIST1, are a frequent cause of coronal craniosynostosis. Nat. Genet..

[194] Davis E.E., Balasubramanian R., Kupchinsky Z.A., Keefe D.L., Plummer L., Khan K., Meczekalski B., Heath K.E., Lopez-Gonzalez V., Ballesta-Martinez M.J. (2020). TCF12 haploinsufficiency causes autosomal dominant Kallmann syndrome and reveals network-level interactions between causal loci. Hum. Mol. Genet..

[195] Uittenbogaard M., Chiaramello A. (2002). Expression of the bHLH transcription factor Tcf12 (ME1) gene is linked to the expansion of precursor cell populations during neurogenesis. Gene Expr. Patterns.

[196] Blümel R., Zink M., Klopocki E., Liedtke D. (2019). On the traces of tcf12: Investigation of the gene expression pattern during development and cranial suture patterning in zebrafish (*Danio rerio*). PLoS ONE.

[197] Quaynor S.D., Bosley M.E., Duckworth C.G., Porter K.R., Kim S.-H., Kim H.-G., Chorich L.P., Sullivan M.E., Choi J.-H., Cameron R.S. (2016). Targeted next generation sequencing approach identifies eighteen new candidate genes in normosmic hypogonadotropic hypogonadism and Kallmann syndrome. Mol. Cell. Endocrinol..

[198] Johnston J.J., Sapp J., Turner J.T., Amor D., Aftimos S., Aleck K.A., Bocian M., Bodurtha J.N., Cox G.F., Curry C.J. (2010). Molecular analysis expands the spectrum of phenotypes associated with GLI3 mutations. Hum. Mutat..

[199] Taroc E.Z.M., Naik A.S., Lin J.M., Peterson N.B., Keefe D.L., Genis E., Fuchs G., Balasubramanian R., Forni P.E. (2019). Gli3 Regulates Vomeronasal Neurogenesis, Olfactory Ensheathing Cell Formation, and GnRH-1 Neuronal Migration. J. Neurosci..

[200] Tischfield M.A., Baris H.N., Wu C., Rudolph G., Van Maldergem L., He W., Chan W.-M., Andrews C., Demer J.L., Robertson R. (2010). Human TUBB3 Mutations Perturb Microtubule Dynamics, Kinesin Interactions, and Axon Guidance. Cell.

[201] Chew S., Balasubramanian R., Chan W.-M., Kang P., Andrews C., Webb B.D., MacKinnon S.E., Oystreck D.T., Rankin J., Crawford T.O. (2013). A novel syndrome caused by the E410K amino acid substitution in the neuronal β-tubulin isotype 3. Brain.

[202] Shao Q., Yang T., Huang H., Majumder T., Khot B.A., Khouzani M.M., Alarmanazi F., Gore Y.K., Liu G. (2019). Disease-associated mutations in human TUBB3 disturb netrin repulsive signaling. PLoS ONE.

[203] Latremoliere A., Cheng L., DeLisle M., Wu C., Chew S., Hutchinson E., Sheridan A., Alexandre C., Latremoliere F., Sheu S.-H. (2018). Neuronal-Specific TUBB3 Is Not Required for Normal Neuronal Function but Is Essential for Timely Axon Regeneration. Cell Rep..

[204] Balasubramanian R., Zhang X. (2015). Mechanisms of FGF gradient formation during embryogenesis. Semin. Cell Dev. Biol..

[205] Chan W.K., Price D., Pratt T. (2017). Fgf8 morphogen gradients are differentially regulated by heparan sulphotransferases Hs2st and Hs6st1 in the developing brain. Biol. Open.

[206] Kawauchi S., Shou J., Santos R., Heébert J.M., McConnell S.K., Mason I., Calof A.L. (2005). Fgf8 expression defines a morphogenetic center required for olfactory neurogenesis and nasal cavity development in the mouse. Development.

[207] Falardeau J., Chung W., Beenken A., Raivio T., Plummer L., Sidis Y., Jacobson-Dickman E.E., Eliseenkova A.V., Ma J., Dwyer A. (2008). Decreased FGF8 signaling causes deficiency of gonadotropin-releasing hormone in humans and mice. J. Clin. Investig..

[208] Chung W.C.J., Moyle S.S., Tsai P.-S. (2008). Fibroblast Growth Factor 8 Signaling through Fibroblast Growth Factor Receptor 1 Is Required for the Emergence of Gonadotropin-Releasing Hormone Neurons. Endocrinology.

[209] Gill J.C., Moenter S.M., Tsai P.-S. (2004). Developmental Regulation of Gonadotropin-Releasing Hormone Neurons by Fibroblast Growth Factor Signaling. Endocrinology.

[210] Forni P.E., Bharti K., Flannery E.M., Shimogori T., Wray S. (2013). The Indirect Role of Fibroblast Growth Factor-8 in Defining Neurogenic Niches of the Olfactory/GnRH Systems. J. Neurosci..

[211] Pitteloud N., Acierno J.S., Meysing A., Eliseenkova A.V., Ma J., Ibrahimi O.A., Metzger D.L., Hayes F.J., Dwyer A.A., Hughes V.A. (2006). Mutations in fibroblast growth factor receptor 1 cause both Kallmann syndrome and normosmic idiopathic hypogonadotropic hypogonadism. Proc. Natl. Acad. Sci. USA.

[212] Simonis N., Migeotte I., Lambert N., Perazzolo C., De Silva D.C., Dimitrov B., Heinrichs C., Janssens S., Kerr B., Mortier G. (2013). FGFR1mutations cause Hartsfield syndrome, the unique association of holoprosencephaly and ectrodactyly. J. Med Genet..

[213] Villanueva C., Jacobson-Dickman E., Xu C., Manouvrier S., Dwyer A.A., Sykiotis G.P., Beenken A., Liu Y., Tommiska J., Hu Y. (2014). Congenital hypogonadotropic hypogonadism with split hand/foot malformation: A clinical entity with a high frequency of FGFR1 mutations. Genet. Med..

[214] Miraoui H., Dwyer A., Sykiotis G., Plummer L., Chung W., Feng B., Beenken A., Clarke J., Pers T., Dworzynski P. (2013). Mutations in FGF17, IL17RD, DUSP6, SPRY4, and FLRT3 Are Identified in Individuals with Congenital Hypogonadotropic Hypogonadism. Am. J. Hum. Genet..

[215] Men M., Wang X., Wu J., Zeng W., Jiang F., Zheng R., Li J.-D. (2020). Prevalence and associated phenotypes of DUSP6, IL17RD and SPRY4 variants in a large Chinese cohort with isolated hypogonadotropic hypogonadism. J. Med. Genet..

[216] Josso N., di Clemente N., Gouédard L. (2001). Anti-Müllerian hormone and its receptors. Mol. Cell. Endocrinol..

[217] Josso N., Racine C., di Clemente N., Rey R.A., Xavier F. (1998). The role of anti-Müllerian hormone in gonadal development. Mol. Cell. Endocrinol..

[218] Behringer R.R., Finegold M.J., Cate R.L. (1994). Müllerian-inhibiting substance function during mammalian sexual development. Cell.

[219] Cimino I., Casoni F., Liu X., Messina A., Parkash J., Jamin S., Catteau-Jonard S., Collier F., Baroncini M., Dewailly D. (2016). Novel role for anti-Müllerian hormone in the regulation of GnRH neuron excitability and hormone secretion. Nat. Commun..

[220] Malone S.A., Papadakis G.E., Messina A., Mimouni N.E.H., Trova S., Imbernon M., Allet C., Cimino I., Acierno J., Cassatella D. (2019). Defective AMH signaling disrupts GnRH neuron development and function and contributes to hypogonadotropic hypogonadism. eLife.

[221] Cannarella R., Paganoni A., Cicolari S., Oleari R., Condorelli R., La Vignera S., Cariboni A., Calogero A., Magni P. (2021). Anti-Müllerian Hormone, Growth Hormone, and Insulin-Like Growth Factor 1 Modulate the Migratory and Secretory Patterns of GnRH Neurons. Int. J. Mol. Sci..

[222] Prosser H.M., Bradley A., Caldwell M.A. (2007). Olfactory bulb hypoplasia in Prokr2 null mice stems from defective neuronal progenitor migration and differentiation. Eur. J. Neurosci..

[223] Matsumoto S.-I., Yamazaki C., Masumoto K.-H., Nagano M., Naito M., Soga T., Hiyama H., Matsumoto M., Takasaki J., Kamohara M. (2006). Abnormal development of the olfactory bulb and reproductive system in mice lacking prokineticin receptor PKR2. Proc. Natl. Acad. Sci. USA.

[224] Dodé C., Teixeira L., Levilliers J., Fouveaut C., Bouchard P., Kottler M.-L., Lespinasse J., Lienhardt-Roussie A., Mathieu M., Moerman A. (2006). Kallmann Syndrome: Mutations in the Genes Encoding Prokineticin-2 and Prokineticin Receptor-2. PLoS Genet..

[225] Cole L.W., Sidis Y., Zhang C., Quinton R., Plummer L., Pignatelli D., Hughes V.A., Dwyer A., Raivio T., Hayes F.J. (2008). Mutations inProkineticin 2andProkineticin receptor 2genes in Human Gonadotrophin-Releasing Hormone Deficiency: Molecular Genetics and Clinical Spectrum. J. Clin. Endocrinol. Metab..

[226] Cox K.H., Oliveira L.M.B., Plummer L., Corbin B., Gardella T., Balasubramanian R., Crowley W.F. (2017). Modeling mutant/wild-type interactions to ascertain pathogenicity of PROKR2 missense variants in patients with isolated GnRH deficiency. Hum. Mol. Genet..

[227] Schwarting G.A., Kostek C., Bless E.P., Ahmad N., Tobet S.A. (2001). Deleted in Colorectal Cancer (DCC) Regulates the Migration of Luteinizing Hormone-Releasing Hormone Neurons to the Basal Forebrain. J. Neurosci..

[228] Schwarting G.A., Raitcheva D., Bless E.P., Ackerman S.L., Tobet S. (2004). Netrin 1-mediated chemoattraction regulates the migratory pathway of LHRH neurons. Eur. J. Neurosci..

[229] Bouilly J., Messina A., Papadakis G.E., Cassatella D., Xu C., Acierno J., Tata B., Sykiotis G., Santini S., Sidis Y. (2017). DCC/NTN1 complex mutations in patients with congenital hypogonadotropic hypogonadism impair GnRH neuron development. Hum. Mol. Genet..

[230] Kotan L.D., Cooper C., Darcan Ş., Carr I.M., Özen S., Yan Y., Hamedani M.K., Gürbüz F., Mengen E., Turan I. (2016). Idiopathic Hypogonadotropic Hypogonadism Caused by Inactivating Mutations in SRA1. J. Clin. Res. Pediatr. Endocrinol..

[231] Bamshad M., Lin R.C., Law D.J., Watkins W.S., Krakowiak P.A., Moore M.E., Franceschini P., Lala R., Holmes L.B., Gebuhr T.C. (1997). Mutations in human TBX3 alter limb, apocrine and genital development in ulnar-mammary syndrome. Nat. Genet..

[232] Galazzi E., Duminuco P., Moro M., Guizzardi F., Marazzi N., Sartorio A., Avignone S., Bonomi M., Persani L., Bonati M.T. (2018). Hypogonadotropic hypogonadism and pituitary hypoplasia as recurrent features in Ulnar-Mammary syndrome. Endocr. Connect..

[233] Eriksson K.S., Mignot E. (2009). T-box 3 is expressed in the adult mouse hypothalamus and medulla. Brain Res..

[234] Huisman C., Cho H., Brock O., Lim S.J., Youn S.M., Park Y., Kim S., Lee S.-K., Delogu A., Lee J.W. (2019). Single cell transcriptome analysis of developing arcuate nucleus neurons uncovers their key developmental regulators. Nat. Commun..

[235] Quarta C., Fisette A., Xu Y., Colldén G., Legutko B., Tseng Y.-T., Reim A., Wierer M., De Rosa M.C., Klaus V. (2019). Functional identity of hypothalamic melanocortin neurons depends on Tbx3. Nat. Metab..

[236] Zhu Z., Han X., Li Y., Han C., Deng M., Zhang Y., Shen Q., Cao Y., Li Z., Wang X. (2019). Identification of ROBO1/2 and SCEL as candidate genes in Kallmann syndrome with emerging bioinformatic analysis. Endocrine.

[237] Cariboni A., Andrews W.D., Memi F., Ypsilanti A.R., Zelina P., Chedotal A., Parnavelas J.G. (2012). Slit2 and Robo3 modulate the migration of GnRH-secreting neurons. Development.

[238] Taroc E.Z., Lin J.M., Tulloch A.J., Jaworski A., Forni P.E. (2019). GnRH-1 Neural Migration from the Nose to the Brain Is Independent From Slit2, Robo3 and NELL2 Signaling. Front. Cell. Neurosci..

[239] Oleari R., André V., Lettieri A., Tahir S., Roth L., Paganoni A., Eberini I., Parravicini C., Scagliotti V., Cotellessa L. (2020). A Novel SEMA3G Mutation in Two Siblings Affected by Syndromic GnRH Deficiency. Neuroendocrinology.

[240] Jee Y.H., Won S., Lui J.C., Jennings M., Whalen P., Yue S., Temnycky A.G., Barnes K.M., Cheetham T., Boden M.G. (2020). DLG2 variants in patients with pubertal disorders. Genet. Med..

[241] Barraud S., Delemer B., Poirsier-Violle C., Bouligand J., Mérol J.-C., Grange F., Higel-Chaufour B., Decoudier B., Zalzali M., Dwyer A. (2020). Congenital Hypogonadotropic Hypogonadism with Anosmia and Gorlin Features Caused by a PTCH1 Mutation Reveals a New Candidate Gene for Kallmann Syndrome. Neuroendocrinology.

[242] Messina A., Langlet F., Chachlaki K., Roa J., Rasika S., Jouy N., Gallet S., Gaytan F., Parkash J., Tena-Sempere M. (2016). A microRNA switch regulates the rise in hypothalamic GnRH production before puberty. Nat. Neurosci..

[243] Ahmed K., Lapierre M.P., Gasser E., Denzler R., Yang Y., Ruelicke T., Kero J., Latreille M., Stoffel M. (2017). Loss of microRNA-7a2 induces hypogonadotropic hypogonadism and infertility. J. Clin. Investig..

[244] Iivonen A.-P., Känsäkoski J., Vaaralahti K., Raivio T. (2019). Screening for mutations in selected miRNA genes in hypogonadotropic hypogonadism patients. Endocr. Connect..

[245] Stamou M., Ng S.-Y., Brand H., Wang H., Plummer L., Best L., Havlicek S., Hibberd M., Khor C.C., Gusella J. (2019). A Balanced Translocation in Kallmann Syndrome Implicates a Long Noncoding RNA, RMST, as a GnRH Neuronal Regulator. J. Clin. Endocrinol. Metab..

